# Design Trade-Offs between Target Binding and Self-Association Revealed by *De Novo* SARS-CoV‑2 Binders

**DOI:** 10.1021/acs.jcim.6c02394

**Published:** 2026-10-27

**Authors:** Lourdes Carcelén, Daniel Peñas-Utrilla, Diego S. Ferrero, Cristina Ferrer-Orta, Marta Nadal, Francesco Colizzi, Juana C. Muñoz, Marta Taulés, Núria Verdaguer, Enrique Marcos

**Affiliations:** † Protein Design and Modeling Lab, Department of Structural Biology, Molecular Biology Institute of Barcelona (IBMB), CSIC, Baldiri Reixac 10, 08028 Barcelona, Spain; ‡ Virus and Large Biological Complexes, Department of Structural Biology, Molecular Biology Institute of Barcelona (IBMB), CSIC, Baldiri Reixac 15, 08028 Barcelona, Spain; § Molecular Ocean Lab, Institute for Advanced Chemistry of Catalonia (IQAC), CSIC, Jordi Girona 18-26, 08034 Barcelona, Spain; ∥ Department of Marine Biology and Oceanography, Institut de Ciències del Mar (ICM), CSIC, Passeig Marítim de la Barceloneta 37-49, 08003 Barcelona, Spain; ⊥ Molecular Interactions Analysis Unit, Centres Científics i Tecnològics (CCIT), Universitat de Barcelona (UB), Barcelona 08028, Spain

## Abstract

*De novo* protein binders have emerged as a powerful platform for molecular recognition, yet achieving high affinity while maintaining favorable biophysical properties remains a major challenge. Here, using the SARS-CoV-2 receptor-binding domain (RBD) as a model target, we generated two *de novo* protein binder design campaigns that deliberately imposed different trade-offs between binding and monomeric behavior, and benchmarked them against a set of experimentally validated binders. Through integrated experimental and computational analyses, we show that binder design is constrained by two distinct failure modes that require different optimization strategies. None of the 22 original designs showed measurable binding to the RBD, with one class remaining monomeric but failing to achieve sufficient target binding, while the other had more competent binding interfaces but exhibited excessive surface hydrophobicity that promoted self-association. By integrating AlphaFold-based predictions, molecular dynamics simulations, docking analyses, and surface aggregation propensity (SAP) calculations, we establish complementary computational signatures that distinguish these failure modes and reveal their underlying structural determinants. Guided by these insights, we prospectively redesigned a representative failed binder by reducing noninterface hydrophobicity while preserving folding and binding interface quality. Experimental characterization by SEC-MALS, surface plasmon resonance, and circular dichroism confirmed that the redesigned variants were stable monomers and acquired low μM binding affinities. Together, our results define the mechanistic trade-offs between binding and self-association in *de novo* protein binder design and provide a framework for diagnosing and rationally overcoming common design failure modes.

## Graphical abstract

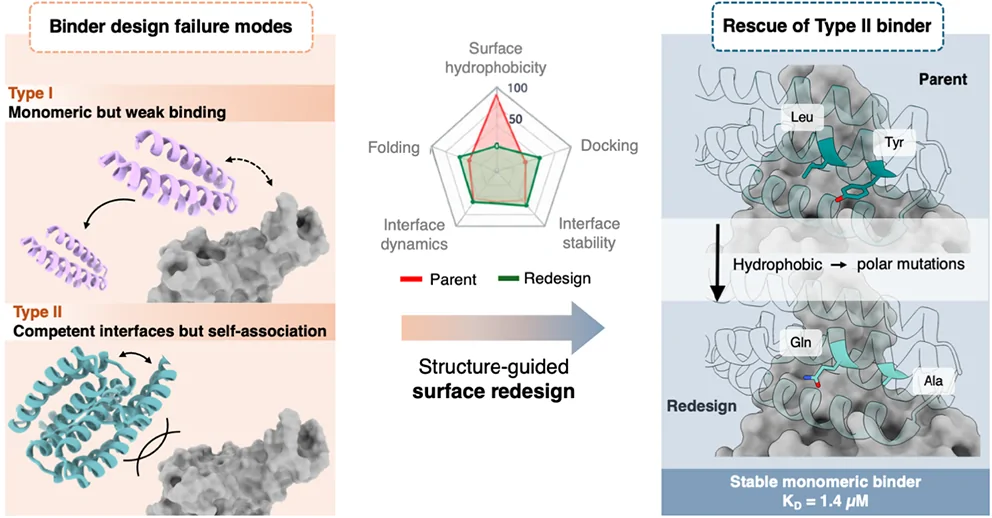

## Introduction

Computational protein binder design has become a powerful approach for engineering proteins that target specific proteins for applications in therapeutics, biosensing, and affinity reagents in biological research. In recent years, the *de novo* design of protein binders targeting proteins of choice has advanced rapidly, driven in large part by breakthroughs in deep learning-based methods for protein structure prediction and design.^1–4^ Earlier strategies, largely built on the Rosetta framework,^5^ relied on generating large libraries of small and compact protein backbones—often helical bundles—followed by docking these scaffolds onto predefined target epitopes, optimizing amino acid sequences through side chain rotamer sampling combined with physics-based energy functions and filtering the resulting designs based on folding and binding interface quality metrics.^6,7^ However, the limited accuracy of energy functions, incomplete conformational sampling, and suboptimal filtering criteria posed significant challenges. As a result, experimental success rates were typically low, requiring extensive experimental screening followed by iterative affinity maturation to obtain high-affinity candidates, with success rates generally below 1% or 0.01% depending on the target.^6,7^ Recently, AI-driven design methods have transformed this landscape. Modern tools enable the rapid generation of high-quality protein backbones alongside sequence optimization tailored to specific epitopes through design pipelines integrating methods such as RFdiffusion,^4^ ProteinMPNN^8^ and AlphaFold^9–11^ methods. These advances now allow the design of stable binders targeting specific epitopes with substantially improved success rates, often yielding medium- to high-affinity candidates with a lower dependence on high-throughput experimental validation.

Despite these advances, a major challenge in *de novo* protein binder design remains the limited mechanistic understanding of why computational designs succeed or fail, which hinders the effective filtering and prioritization of candidate binders for experimental validation. More fundamentally, experimental success depends on the simultaneous optimization of multiple competing properties. First, binders must reliably fold into the intended three-dimensional structure, adopting conformations compatible with target recognition. Second, they must establish shape- and chemically complementary interactions that yield a sufficiently strong and stable protein–protein complex. Third, they must exhibit adequate monomeric behavior to present the designed binding interface while avoiding self-association, oligomerization, or aggregation. Consequently, identifying the most promising candidates for experimental validation requires balancing several competing objectives, which are typically approximated using complementary physics-based metrics (e.g., Rosetta binding energies or shape complementarity) together with AI-based predictors (e.g., AlphaFold confidence scores and structural agreement between the design model and predicted structure). In most practical settings, only a small fraction of computational designs can be experimentally evaluated, making accurate prioritization essential for maximizing experimental success. Beyond improving the efficiency and accessibility of protein binder design, increasing experimental success rates would also deepen our understanding of the fundamental principles governing protein–protein interactions. However, despite the growing number of experimentally characterized *de novo* binders, relatively few studies have systematically investigated the mechanisms underlying computational design failures, limiting our understanding of the distinct failure modes that govern experimental success and our ability to derive general design principles from both successful and unsuccessful candidates.

Here, we investigated how folding, binding, and monomeric behavior are interconnected in *de novo* protein binder design, and how imbalances among these properties give rise to distinct modes of experimental failure. Using the SARS-CoV-2 receptor-binding domain (RBD) as a model target, we generated *de novo* binders spanning a range of interface sizes and surface hydrophobicities and characterized them experimentally using low-throughput solution-based assays. Retrospective analyses integrating physics-based modeling, molecular dynamics simulations, and deep learning structure prediction identified two contrasting failure modes associated with insufficient interface stability or excessive self-association driven by exposed hydrophobicity. Guided by these mechanistic insights, we prospectively redesigned a previously unsuccessful binder to improve monomeric behavior while preserving binding competence, ultimately rescuing a highly stable monomeric binder with low micromolar affinity. Together, our results demonstrate that successful *de novo* protein binder design depends on achieving an appropriate balance between folding, binding, and monomeric behavior.

## Results

### Computational Design of Protein Binders

Beyond its biomedical relevance for antiviral therapeutic development, the receptor-binding domain (RBD) of SARS-CoV-2 provides an ideal model system for studying *de novo* protein binder design because a large number of computationally designed binders have been experimentally characterized and can serve as positive controls. During the COVID-19 pandemic, extensive structural and biochemical studies established the molecular basis of RBD recognition by the human ACE2 receptor,^12^ neutralizing antibodies,^13–16^ and *de novo*-designed protein binders.^7,17^ In particular, Cao et al.^7^ generated thousands of *de novo* binders using Rosetta through two complementary design strategies: grafting ACE2-derived binding helices or constructing entirely new binding interfaces from scratch. Despite this large computational effort, only a small fraction of designs (<0.1%) exhibited detectable binding activity in yeast surface display assays. Subsequent affinity maturation of the initial hits produced a series of high-affinity binders (picomolar to nanomolar) with high thermostability. Importantly, the computational models of these optimized binders were made publicly available, and cryo-electron microscopy structures of representative binder–RBD complexes confirmed the accuracy of the design models. Therefore, for this study, we selected the ten most potent binders (LCB1–8 and AHB1–2) as a positive control set representing experimentally validated examples of successful *de novo* protein binders (Figure 1).

**Figure 1:**
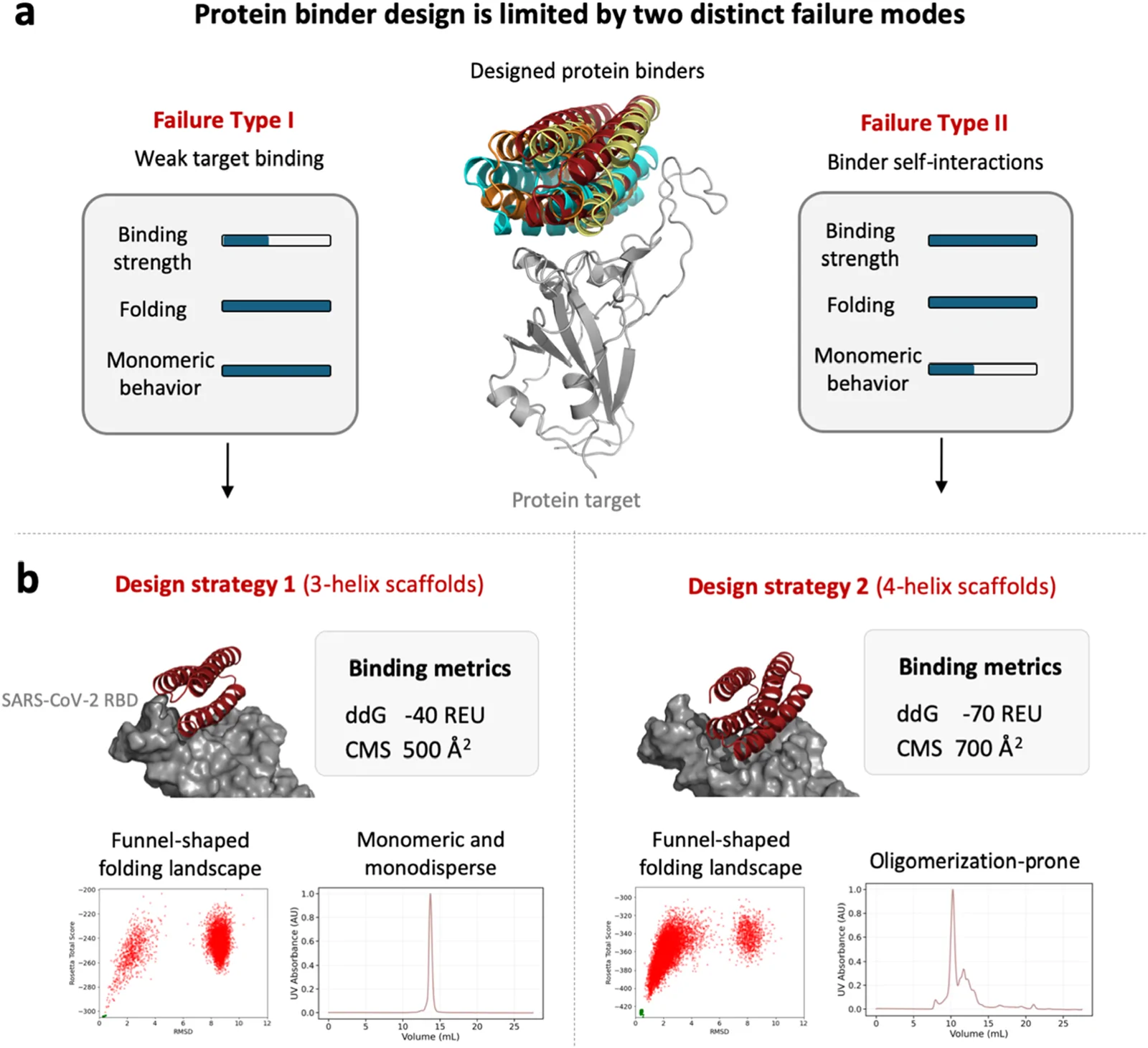
Competing objectives underlying *de novo* binder design. (a) Successful binders must balance target-binding affinity, folding stability, and monomeric behavior. Two major failure modes are illustrated. Failure Type I arises when folding and monomeric behavior are favored at the expense of target binding, resulting in weak binders. Failure Type II occurs when strong intermolecular interactions are accompanied by self-association, compromising monomeric behavior. These observations motivated two successive design rounds aimed at improving distinct aspects of binder performance. (b) Characteristics of binders generated in the two design rounds. Round 1 employed compact three-helix scaffolds, whereas Round 2 incorporated larger four-helix scaffolds to enhance the binding interface. The Rosetta metrics ddG and CMS correspond to the predicted binding free energy and contact molecular surface area,^6^ respectively. In both rounds, designs were optimized for foldability, as evidenced by funnel-shaped energy landscapes obtained from Rosetta *ab initio* structure prediction (bottom left panels). Representative binding models against the SARS-CoV-2 receptor-binding domain (RBD) are shown together with folding and biophysical characterization data. SEC-MALS analysis revealed a predominantly monomeric species for the representative Round 1 design (R1–10), whereas the representative Round 2 design (R2–5) exhibited a broader elution profile consistent with increased oligomerization and sample heterogeneity. Design models and experimental data are shown for representative binders from each design round.

To investigate how different design priorities influence experimental success, we generated protein binders through two contrasting design strategies that imposed distinct trade-offs between monomeric behavior and interface strength. In the first design round, we focused on maximizing folding stability by engineering binders that mimic helix α1 of ACE2, which mediates most of the interactions with the SARS-CoV-2 RBD in the crystal structure of the ACE2–RBD complex (PDB ID: 6M0J).^12^ This recognition motif was embedded within a well-folded three-helix bundle scaffold, a robust topology that has been widely and successfully used in *de novo* protein design. In the second design round, we prioritized increasing interface strength by expanding the binding surface relative to the first-round designs. To this end, we designed four-helix binders that not only mimic ACE2 helix α1 but also extend interactions toward a second RBD epitope recognized by the ACE2 β-hairpin, specifically the helical loop connecting strands β6 and β7 (RBD residues 482–491).

For both design strategies, protein backbones were generated using Rosetta fragment assembly guided by blueprint files specifying α-helical segments connected by short loops.^18^ To preserve the native binding geometry, ACE2 helix α1 extracted from the ACE2–RBD crystal structure was incorporated as a fixed structural motif during backbone generation, while its C-terminus was progressively truncated to remove noncontacting residues and minimize scaffold size. Systematic sampling of helix (18–24) and loop (2–4) lengths generated thousands of compact backbone conformations optimized for both folding stability and presentation of shape-complementary binding interfaces. These backbones subsequently underwent combinatorial sequence optimization in Rosetta^19^ and were ranked according to complementary metrics describing folding stability, core packing, interface quality, and local sequence–structure compatibility. The highest-ranking designs were further evaluated using *ab initio* Rosetta folding simulations,^20^ and only models displaying well-defined funnel-shaped energy landscapes were selected for experimental characterization (Figures S1 and S2). This pipeline yielded 22 candidate binders for experimental testing (11 from each design round; Table S1). As intended, the two design strategies produced binders with distinct structural characteristics. First-round designs were compact proteins of 70–80 amino acids that formed relatively small interfaces (median values of ∼200 interface contacts, ∼550 Å^2^ contact molecular surface, and interface ddG scores of approximately −40 Rosetta energy units). In contrast, second-round binders were substantially larger (106 amino acids) and formed more extensive interfaces (median values of ∼230 contacts, ∼700 Å^2^ contact molecular surface, and ddG scores of approximately −70 Rosetta energy units), consistent with the objective of strengthening target interactions.

Synthetic genes encoding the 22 designed binders were recombinantly expressed in *Escherichia coli*, and the proteins were purified using affinity and size-exclusion chromatography (SEC). All first-round designs were well-expressed, soluble, and predominantly monomeric by SEC coupled with multiangle light scattering (SEC-MALS) (Figure S3), but exhibited very weak or no detectable binding to the target based on ELISA or surface plasmon resonance (SPR) assays (Figures S4 and S5). In contrast, second-round designs, which were optimized to maximize binding interface contacts while maintaining a stable scaffold, showed poorer biophysical behavior: six either failed to express or expressed insolubly, while the remaining five were soluble and could be purified but predominantly formed dimers or higher-order oligomers by SEC-MALS (Figure S6). Although all designed binders ultimately failed experimentally, the distinct biophysical behaviors observed in the two rounds suggest that they failed for different reasons, likely arising from imbalances between binder folding, binding and monomeric behavior.

### Retrospective Analysis of the Designed Binders

To better understand the factors underlying these failures, we performed a comprehensive analysis of folding, binding, and monomeric behavior by integrating additional physics-based methods with recent deep learning tools for protein structure prediction. We also compared the predicted metrics of our designs against a control set of high-affinity *de novo* protein binders from Cao et al.,^7^ using the top 10 available design models from that study (LCB1–8, and AHB1–2).

We began by analyzing the monomeric folding of the binders using AlphaFold2 (AF2) structure prediction^11^ (Figure 2a), and evaluated the predictions based on confidence metrics (pLDDT and pTM) and the structural recapitulation of the design models (RMSD). For both rounds, designs were predicted with high confidence and closely matched their computational models, with mean pLDDT scores of 90–93, pTM scores of 0.7–0.8 and Cα-RMSD values of 1.1–1.3 Å. No substantial differences were observed relative to the control set of active binders, which showed similarly accurate predictions. These results are consistent with the prior validation of the designs through Rosetta *ab initio* folding simulations and further support the conclusion that binders from both rounds were generally well-designed at the monomeric folding level.

**Figure 2:**
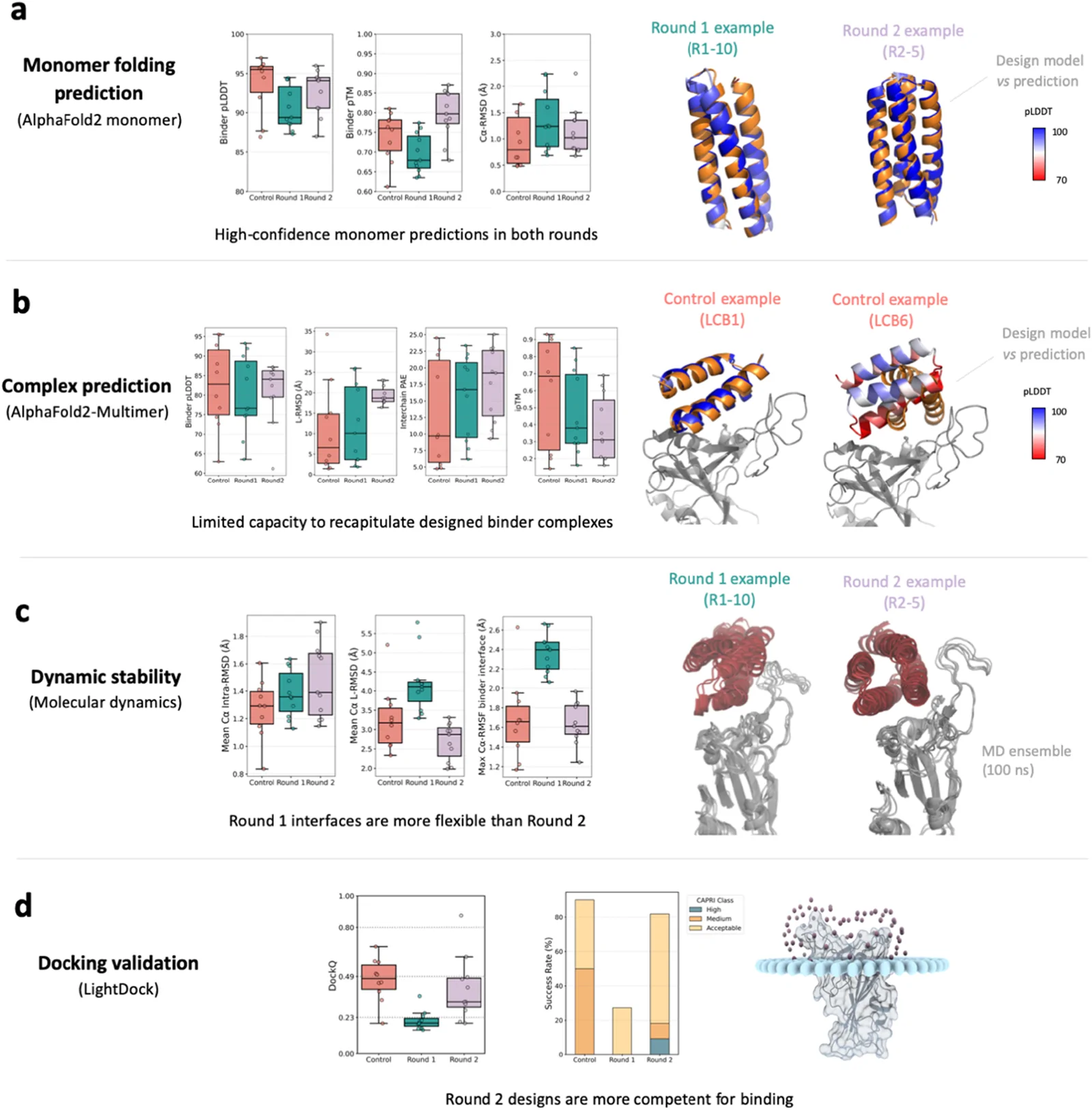
Retrospective analysis of folding quality, binding interface stability, and docking performance of the designed binders. (a) AlphaFold2 monomer predictions for binders from the control set and two successive design rounds (Round 1 and Round 2). Boxplots show binder confidence (pLDDT), predicted TM-score (pTM), and Cα-RMSD relative to the design model. Representative designs from Round 1 (R1–10) and Round 2 (R2–5) are shown on the right, with the design model (orange) overlaid with the top-ranked AlphaFold2 prediction colored by pLDDT. (b) AlphaFold2-Multimer evaluation of binder–target complexes. Distributions of binder pLDDT, ligand RMSD (L-RMSD), interchain predicted aligned error (PAE), and ipTM are shown for each design set. Representative predictions for the control binders LCB1 and LCB6 are displayed, with the target protein shown in gray and the binder colored by prediction confidence and overlaid with the design model (orange). Whereas the LCB1 complex is recovered with high accuracy, the predicted LCB6 complex deviates substantially from the design model, highlighting the difficulty of accurately predicting high-affinity binder–target complexes in this system using AlphaFold2-Multimer alone. (c) Molecular dynamics simulations of the designed protein complexes. Boxplots summarize the time-averaged intramolecular Cα-RMSD of the binder, ligand Cα-RMSD relative to the target (capturing both conformational and rigid-body motions), and the maximum Cα-RMSF of binder interface residues across simulation replicas. Representative trajectory ensembles for designs from Round 1 and Round 2 are shown on the right, with the target rendered in gray and the binder ensemble in red. The Round 2 design exhibits a more rigid and well-defined binding interface than the Round 1 design. (d) Membrane-constrained protein–protein docking using LightDock. Right, schematic of the docking setup with the membrane represented as a lipid bilayer. Left, DockQ scores obtained for each design set. Center, docking success rates classified according to CAPRI quality criteria (acceptable, medium, and high quality). Docking results support improvement in interface quality for Round2-designs in comparison with Round1.

Next, we performed cofolding predictions with AlphaFold2-Multimer^10^ (AFM) to evaluate both binder folding and docking orientation relative to the target (Figure 2b). Predicted complexes were assessed using structural accuracy metrics (L-RMSD) and confidence metrics, including pLDDT, ipTM and predicted aligned error (PAE) at the interface. AFM showed limited ability to accurately predict the docking orientation of the active binders, likely due to the lack of coevolutionary information across the designed interfaces. While some active binders, such as LCB1 and LCB3, were predicted with high accuracy, others, including LCB2 and LCB6, showed poor interface confidence and large deviations from the design models. Importantly, predictions for the active binders were slightly better in terms of both confidence and structural similarity to the design models (Figure 2b), but remained insufficiently accurate (with median binder pLDDT values of 83 versus 77–84, ipTM scores of 0.68 versus 0.31–0.38, and L-RMSD values of 6.5 Å versus 10–19 Å) for AFM predictions to reliably discriminate successful from unsuccessful binders. In most cases, AFM accurately predicted monomeric folding but failed to reliably recover the designed binding orientation. Notably, several inactive designs—particularly from round 1—achieved confidence scores comparable to some active binders, such as R1–3 and R1–6.

These limitations of AFM are consistent with previous reports showing that prediction accuracy can vary substantially depending on the system. We next tested whether prediction accuracy for the controls, as well as discrimination between binding and nonbinding proteins, could be improved using the “AF2 initial guess” approach,^1^ which initializes the pair representation from the design model and has been shown to improve predictions for complexes that are mispredicted by AF2. However, in our data set, this approach did not significantly improve the prediction of the controls or the discrimination from nonbinding proteins (Figure S7a). We also performed AlphaFold3^9^ (AF3) predictions using 100 seeds, which yielded improved predictions for the control set, whereas the Round 1 and Round 2 designs generally produced low-confidence predictions (Figure S7b). With the exception of AHB1 and LCB6, the control designs yielded high-confidence predictions that closely matched the corresponding design models, suggesting that the designed interfaces in the Round 1 and Round 2 binders may be less optimal and/or more challenging for AF3 to predict accurately.

To gain deeper insight into the structural stability of the designed binding interfaces, we performed all-atom molecular dynamics (MD) simulations of each binder–RBD complex using three independent 100 ns replicas at 300 K (Figure 2c and Figures S8–S10). To separately assess interface stability and monomer folding, we monitored two complementary metrics throughout the trajectories: (i) L-RMSD, defined as the Cα-RMSD of the binder after superposition onto the receptor, which captures both internal conformational changes and rigid-body motions relative to the target; and (ii) the internal Cα-RMSD of the binder, which reflects the intramolecular flexibility of the binder, independently of its orientation relative to the target. In addition, we calculated per-residue root-mean-square fluctuations (RMSF) for interface residues and used the maximum interface RMSF (maxRMSF) as a complementary measure of interface flexibility. Active binders and second-round inactive designs exhibited comparable L-RMSD values (median 2.03 and 2.32 Å, respectively) and similarly low internal RMSD values (median 1.12–1.16 Å), indicating stable folded structures throughout the simulations. In contrast, first-round designs displayed substantially larger L-RMSD values (median 2.90 Å) while maintaining similarly low internal RMSD values (median 1.31 Å), indicating that the increased deviations primarily arise from larger rigid-body motions of the binder relative to the target rather than from unfolding of the binder itself. Consistent with this interpretation, first-round designs also exhibited greater flexibility at the binding interface, with median maxRMSF values of 2.53 Å compared with 1.73 and 1.72 Å for the active binders and second-round designs, respectively. Together, these results indicate that the poor experimental performance of first-round binders is primarily associated with insufficient stability of the protein–protein interface rather than deficiencies in monomer folding, consistent with the high folding confidence predicted by AlphaFold2 and the stable intramolecular structures observed throughout the MD simulations.

Given the lack of consensus among AlphaFold-based methods in assessing how well the different binders dock to the target in this data set, we next performed a physics-based docking analysis to independently assess the binding competence of the designed interfaces. We used LightDock,^21^ a swarm-based docking algorithm, while restricting sampling to the expected RBD epitope through a spatial constraint that mimics the presence of a membrane. Docking models were ranked using the DFIRE scoring function, and the highest-quality poses were evaluated with DockQ, which integrates interface RMSD, ligand-RMSD, and the fraction of native contacts into a continuous measure of docking accuracy (Figure 2d). Active binders displayed substantially higher docking quality than either group of inactive designs, with a median DockQ score of 0.48 compared with 0.19 for first-round designs and 0.33 for second-round designs. Notably, second-round designs exhibited a broader distribution of scores, with several designs achieving DockQ values comparable to, or even exceeding, those of experimentally active binders. Consistent with these observations, CAPRI classification showed that 9 of the 10 active binders generated at least acceptable-quality docking models, including 5 with medium accuracy. In contrast, only 3 of the 11 first-round designs achieved acceptable-quality models, and none reached medium or high accuracy. Second-round designs performed considerably better, with 7 of the 11 designs producing acceptable-quality models and 2 reaching medium or higher accuracy. Together, the docking and MD analyses consistently indicate that first-round designs possess suboptimal binding interfaces relative to both active binders and second-round designs, as evidenced by poorer docking performance and increased rigid-body fluctuations at the protein–protein interface. In contrast, the relatively high docking quality and stable interface dynamics of many second-round designs suggest that their interfaces are largely competent for target recognition, implying that their lack of experimental activity is more likely explained by defects in monomeric behavior rather than by insufficient interface quality.

We then evaluated the spatial aggregation propensity^22^ (SAP) of all binders. SAP quantifies exposed hydrophobic patches associated with aggregation-prone regions, with lower values indicating greater solubility and monomeric behavior. Active binders and Round 1 designs exhibited similar SAP scores (median 37 and 29; maximum 50 and 45, respectively), but Round 2 designs showed substantially higher values (median 78; maximum 106), consistent with the increased propensity for self-association observed experimentally (Figure 3a). We then decomposed SAP scores into contributions from interface and noninterface residues. Round 1 designs displayed the lowest interface SAP, consistent with their weaker binding interfaces. In contrast, Round 2 designs exhibited elevated SAP across both regions of the protein. While increased interface SAP is consistent with the stronger and more rigid interfaces identified in our previous analyses, the accompanying increase in noninterface SAP indicates excessive hydrophobic surface exposure outside the binding site, which together with the interface SAP, is likely to promote nonspecific oligomerization and aggregation. This behavior may stem from the design of the four-helix bundles, where optimization for folding energetics was not accompanied by a sufficiently strong penalty on exposed hydrophobic patches. In contrast, the active control binders concentrated a larger fraction of their total SAP at the binding interface (median 71%) compared to Round 1 and 2 designs (43–51%), while maintaining very low SAP out of the interface. We note that SAP is an extensive metric that scales with protein length. However, normalization by sequence length showed that the distinguishing feature of second-round designs is not only their higher total SAP per se, but the excessive contribution of noninterface surface hydrophobicity (Figure S11). Representative examples illustrate these distinct hydrophobicity distributions, with Round 2 designs displaying extensive aggregation-prone patches both at and away from the interface, whereas active binders largely restrict such regions to the binding surface (Figure 3b). Together, these results highlight the challenge of optimizing binding affinity, while minimizing self-association—a balance successfully achieved by the active control binders.

**Figure 3:**
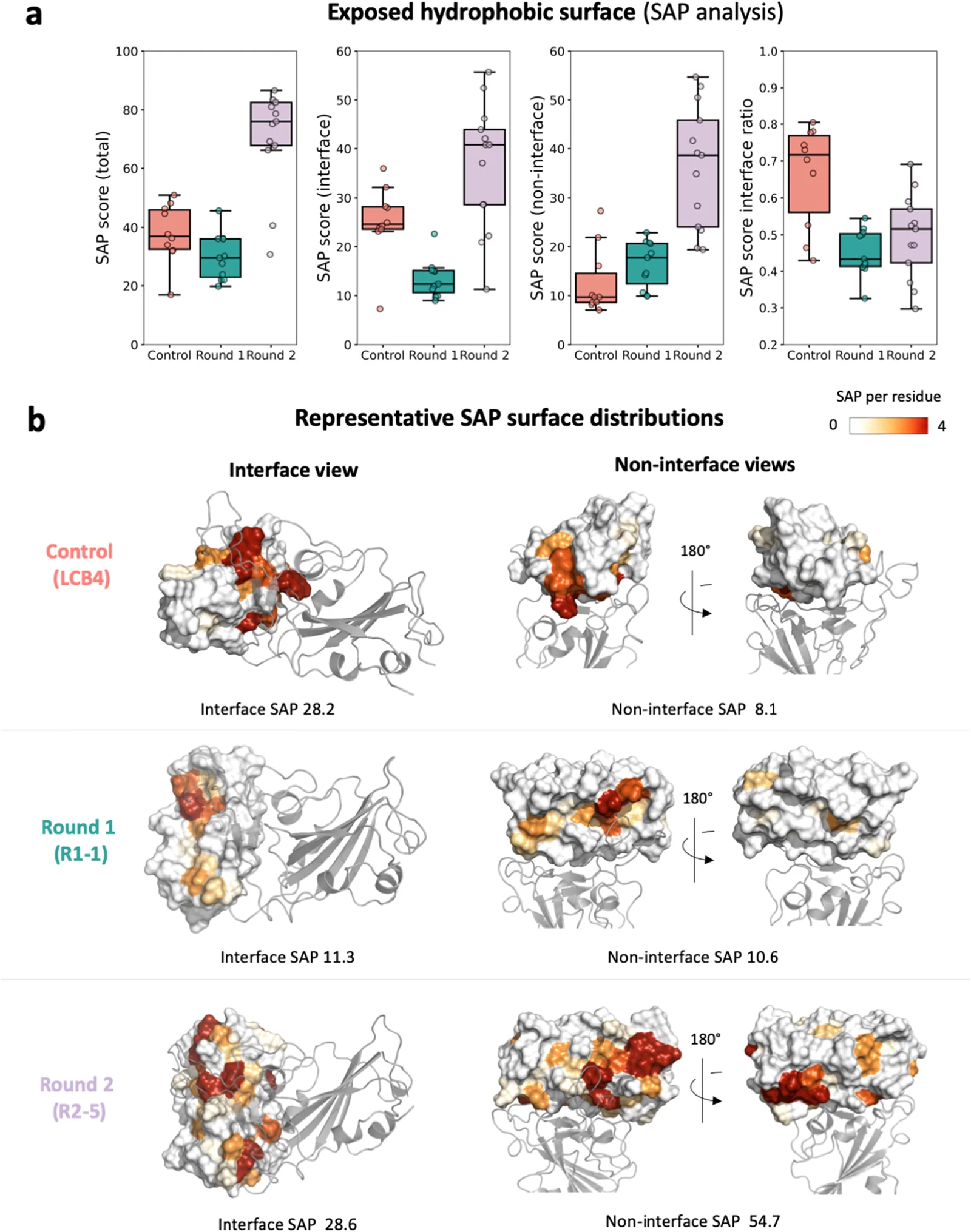
Spatial aggregation propensity (SAP) analysis of the designed binders in the monomeric state. (a) Comparison of total SAP scores across the control, Round 1, and Round 2 design sets (left) shows that Round 2 designs exhibit substantially higher aggregation propensity than both the control and Round 1 binders. Decomposition of the total SAP score into contributions from interface and noninterface residues (middle) reveals that Round 1 designs have markedly lower interface-associated SAP than the other two groups. The fraction of total SAP originating from interface residues (right) indicates that aggregation-prone surface patches are more strongly concentrated at the binding interface in the control set than in either design round. (b) Representative binder–target complexes from each design class colored by per-residue SAP score (increasing from white to red). SAP contributions from interface and noninterface residues are reported for each binder. Left, views centered on the binding interface reveal larger aggregation-prone surface patches in the control (LCB4) and Round 2 (R2–5) designs relative to the representative Round 1 (R1–1) design. Right, views of the solvent-exposed, noninterface binder surface show elevated SAP regions in the R2–5 compared with both LCB4 and R1–1, consistent with the higher overall SAP scores observed for the Round 2 design set.

Overall, these analyses indicate that successful *de novo* protein binders require balanced optimization across multiple structural and functional properties rather than maximization of any single metric. Active binders combined favorable Rosetta interface metrics, high docking quality, stable binding interfaces during molecular dynamics simulations, and surface hydrophobicity that was both sufficiently high and spatially concentrated at the binding interface, reflecting an effective balance between binding strength and monomeric behavior. In contrast, first-round designs prioritized folding stability and monomeric behavior but formed weaker and more flexible interfaces, whereas second-round designs generated larger, better-packed, and dynamically more stable interfaces, as reflected by improved docking performance and MD stability, at the expense of increased surface hydrophobicity and self-association propensity. Together, these results highlight a central trade-off in binder design: excessive optimization of binding interactions can compromise solubility and monomeric behavior, whereas overoptimization of folding and monomeric behavior may limit the binding strength required for productive target engagement.

### Computational Design Rescue of Protein Binders

The computational analyses indicated that second-round binders generally possessed more optimized binding interfaces than first-round designs while maintaining robust folding properties. We therefore hypothesized that selectively reducing surface hydrophobicity while preserving the folding and binding interface quality should decrease self-association and enable target binding. To test this hypothesis, we sought to rescue design R2–6, which was dimeric in solution, by reducing its SAP score to the range observed for experimentally active binders while maintaining favorable computational metrics associated with folding quality and binding competence.

To reduce surface aggregation propensity while preserving the integrity of the folded scaffold and binding interface, we redesigned solvent-exposed residues located in α-helices while leaving loop regions unchanged to minimize perturbations to the backbone structure. The redesign combined two complementary strategies (Figure 4a). First, interface hydrophobic residues predicted by in silico alanine scanning to contribute only marginally to binding were allowed to mutate to polar amino acids, whereas energetically important interface residues remained fixed. Second, highly solvent-exposed residues outside the binding interface were redesigned to favor polar or charged amino acids, thereby reducing exposed hydrophobic patches while promoting favorable surface hydrogen-bonding and salt-bridge interactions. Among the redesigned variants, R2–6.1 was selected for experimental characterization. Surface redesign reduced the total SAP score from 67.7 to 40.6 by lowering aggregation propensity at both the binding interface (28.6 to 20.9) and the noninterface surface (39.1 to 19.6), placing the overall SAP within the range observed for experimentally active binders while remaining higher than that of the first-round designs (see Figure 4a for changes in SAP distribution). Importantly, these changes preserved all computational indicators of binder quality (Figure 4b). AlphaFold2 and molecular dynamics simulations predicted unchanged folding stability, interface binding energy was only minimally affected, and docking performance improved slightly. Although the AF3 prediction of R2–6.1 had a low ipTM score (0.2) and failed to recapitulate the design model (L-RMSD 13.2 Å), similar to the parent R2–6 design (ipTM 0.2; L-RMSD 10.6 Å), we did not use these metrics to filter the redesigns, as our aim was to assess the impact of improved SAP while preserving the quality of the other design metrics. The redesign introduced 17 mutations, including three at the binding interface (L37T, I64E, and M74D) and 14 outside the interface. Five of the noninterface substitutions (I2E, L19Q, Y23A, V57K, and Y102H) directly replaced solvent-exposed hydrophobic residues with high local SAP values by polar or charged amino acids, whereas the remaining mutations primarily reorganized surface electrostatics through the formation of new salt bridges. Notably, the main interface residues identified by alanine scanning—including the hydrophobic residues M47, I63, M66, and F73, together with the electrostatic interactions involving E40, R41, and E44—were intentionally preserved throughout the redesign.

**Figure 4:**
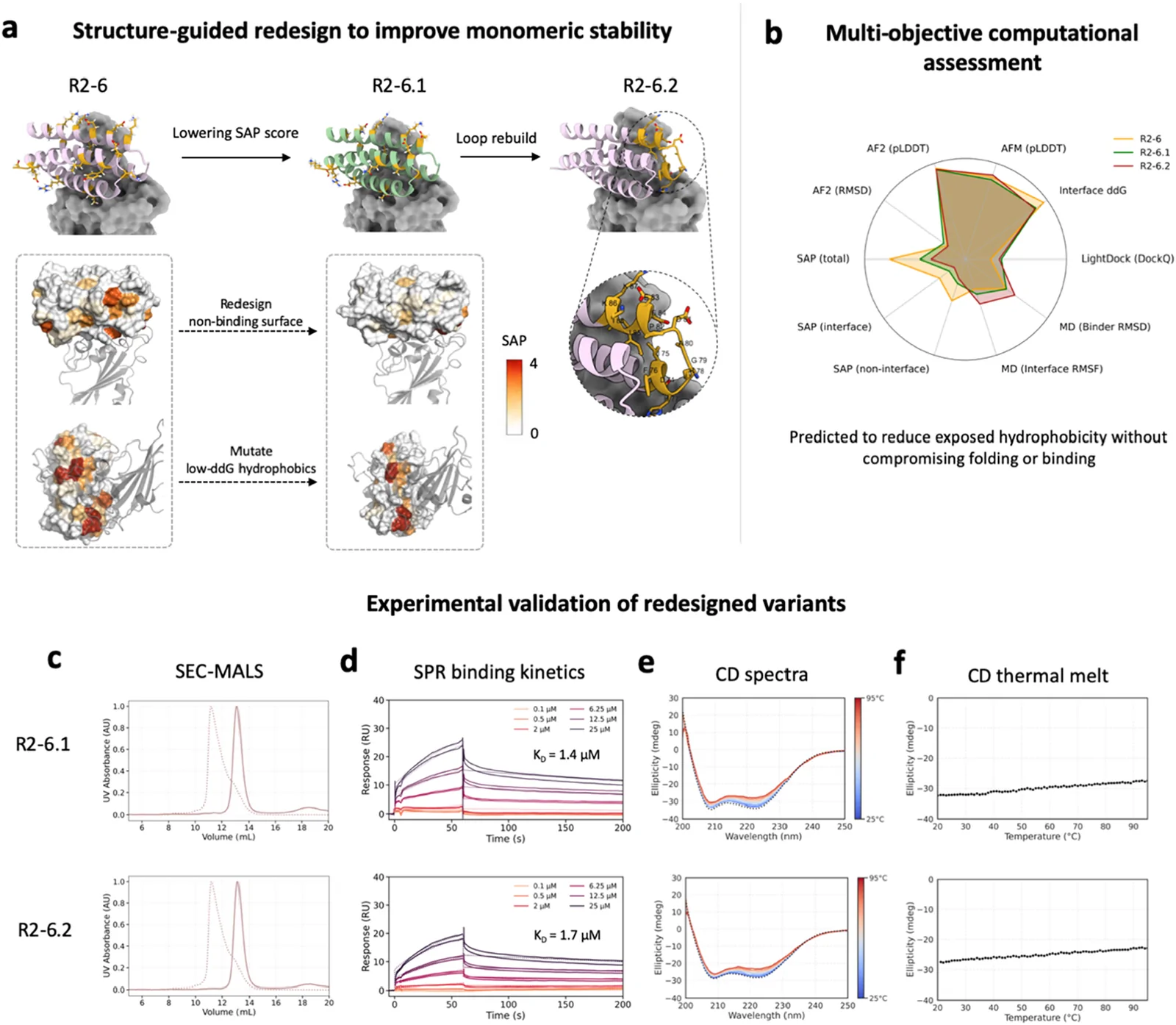
Structure-guided redesign improves monomeric behavior while preserving binding competence. (a) Structure-guided optimization of the Round 2 binder R2–6. First, solvent-exposed hydrophobic residues located either outside the binding interface or at the interface but predicted to make only minor contributions to binding (low-ddG residues) were redesigned to polar amino acids to reduce surface aggregation propensity (SAP), generating variant R2–6.1. SAP surface maps illustrate the resulting reduction in exposed hydrophobic patches. Subsequently, a loop adjacent to the binding interface was rebuilt to optimize folding, producing variant R2–6.2. The inset highlights the remodeled loop region. (b) Multiobjective computational assessment of R2–6 and the two redesigned variants. Integrated computational metrics, including AlphaFold2 monomer confidence, AlphaFold2-Multimer predictions, interface binding energy (ddG), docking performance (DockQ), molecular dynamics stability, and SAP scores, predict that the redesigned variants maintain favorable folding and binding properties while reducing exposed hydrophobicity. (c–f) Experimental validation of the redesigned variants. (c) Size-exclusion chromatography coupled to multiangle light scattering (SEC-MALS) shows that both redesigned variants shift the major elution peak from the dimeric state observed for the parental R2–6 design (dashed lines) to a monomeric state (solid lines) as confirmed by the measured molecular weights (Table S2). (d) Surface plasmon resonance (SPR) sensorgrams demonstrating concentration-dependent binding of both variants to the target. Experimental data (solid lines) were globally fitted to a binding model (dotted lines), yielding the indicated *K*_D_. (e) Circular dichroism (CD) spectra demonstrate that both redesigned variants adopt the expected α-helical secondary structure throughout the 25–95 °C temperature range and recover their initial CD spectra upon cooling back to 25 °C (black dashed line), indicating reversible folding. (f) Thermal melt profiles monitored by CD ellipticity at 220 nm show that both redesigned variants remain structurally stable over the tested temperature range.

To further explore whether local backbone optimization could improve binder performance, we generated a second variant, R2–6.2, by remodeling the loop connecting helices α3 and α4 (Figure 4a, right). In addition to contributing to the structural integrity of the binder, this loop forms tangential contacts with the RBD and therefore represented an attractive target for improving interface packing while preserving the overall binder architecture. The loop sequence was redesigned using consensus profiles derived from naturally occurring loops sharing the same ABEGO torsion pattern, while neighboring residues were simultaneously optimized to accommodate the remodeled backbone. From the resulting designs, we selected a single variant for experimental characterization that maintained SAP scores and molecular dynamics stability comparable to those of R2–6.1 (Figure 4b). As for R2–6.1, AF2 yielded high-confidence predictions for R2–6.2, whereas AF3 failed to accurately reproduce the design model (ipTM 0.75, L-RMSD 24.7 Å). The final design introduced five additional mutations (I32F, F77H, E79G, G80A, and K84E) and remodeled residues 77–82 from the (HH)­AGB­(H) ABEGO pattern to (H)­AGBB­(H), where H denotes the flanking α-helical residues.

Redesigned variants R2–6.1 and R2–6.2 were expressed in *E. coli* using the same protocol as the original designs. Both proteins could be purified by size-exclusion chromatography and remained predominantly monomeric by SEC-MALS (Table S2), confirming that the introduced mutations successfully stabilized the monomeric state (Figure 4c). Surface plasmon resonance (SPR) measurements showed that R2–6.1 bound RBD with a *K*_D_ of 1.4 μM, and the association (*k*_a_ = 1071 M^–1^ s^–1^) and dissociation phases (*k*_d_ = 0.001 s^–1^) were well described by a kinetic binding model (Figure 4d), confirming robust target binding. R2–6.2 exhibited comparable binding affinity and kinetic parameters (*K*_D_ = 1.7 μM, *k*_a_ = 856 M^–1^ s^–1^, *k*_d_ = 0.001 s^–1^), indicating that remodeling the α3−α4 loop did not substantially contribute to target binding while demonstrating that the designed binder retained its structural and functional integrity despite local backbone modification. Moreover, circular dichroism spectroscopy confirmed the expected α-helical secondary structure for both variants, which remained folded throughout the 25–95 °C temperature range and recovered their original spectra upon cooling to 25 °C, indicating reversible thermal unfolding without detectable aggregation and demonstrating their high thermostability (Figure 4e,f). Taken together, these results provide experimental validation of the computational framework developed in this study. By identifying and selectively correcting the predicted cause of failure, we rescued a previously unsuccessful binder and generated redesigned variants that combine robust structural stability, monomeric behavior in solution, and consistent low-micromolar target binding.

Having established that excessive surface hydrophobicity was a major issue in the Round 2 designs, we next asked whether this could have resulted from limitations of the Rosetta sequence design protocol (Methods section)—combined with layer-based amino acid restrictions and the ref 2015 scoring function—and whether it could have been mitigated by using the more recent ProteinMPNN with soluble weights, SolubleMPNN,^23^ which generally disfavors solvent-exposed hydrophobic residues. To test this, we redesigned the full sequences of the 11 Round 2 designs using SolubleMPNN, generating 10 sequences per design, and calculated the SAP scores of the resulting binders (Figure S12). As a control, we similarly redesigned the Round 1 sequences to determine whether their initially favorable SAP scores were maintained with SolubleMPNN.

The overall distribution of SAP scores for the Round 2 redesigns shifted toward lower values (median of 41 compared with 78 for the original designs), although a substantial fraction of sequences still exhibited relatively high SAP scores (28% with SAP ≥ 50). In contrast, the Round 1 redesigns retained favorable SAP scores within a similar range to those of the original designs (median 28 vs 29). These results suggest that the distinct folding and/or binding properties of the Round 2 designs may make it more difficult to balance the burial of hydrophobic residues with the requirements for core stability and favorable binding interactions. Overall, these findings indicate that modern sequence-design tools can mitigate, but not fully eliminate, the problem of exposed hydrophobicity derived from interface design. Careful assessment of surface hydrophobicity therefore remains important to avoid the detrimental effects on solution behavior and binding competence observed here.

## Discussion

Our study demonstrates that successful *de novo* protein binder design is fundamentally a multiobjective optimization problem in which folding, binding, and monomeric behavior must be optimized simultaneously. An effective binder must adopt a well-folded monomeric structure, remain stable and well behaved in solution, and establish sufficiently strong interactions with its target. Through two independent design campaigns, we identified two distinct failure modes that arise from different imbalances among these objectives. Round 1 designs favored folding and monomeric behavior at the expense of target binding. Conversely, Round 2 designs favored target binding at the expense of monomeric behavior, resulting in reduced expression and increased oligomerization driven by excessive surface hydrophobicity. Although these failure modes originate from opposite optimization biases, both ultimately prevent the generation of robust protein binders. Importantly, our study focuses on trade-offs between binding and monomeric behavior after stringent folding validation. All candidate binders were filtered using *ab initio* folding predictions before experimental characterization, effectively minimizing failures arising from poorly folded scaffolds. A third failure mode in *de novo* binder design involves optimizing target interactions at the expense of folding, producing proteins with poorly packed cores, disordered loops, or unstable folds. Although this failure mode was intentionally excluded from the present work through stringent folding validation, it illustrates that binder design may fail through multiple independent optimization imbalances rather than through a single dominant mechanism.

No single computational method consistently captured all aspects of binder quality. Instead, combining deep learning–based predictors with physics-based simulations provided complementary information that enabled the two failure modes to be distinguished. AlphaFold2 reliably evaluated monomer folding quality, whereas AFM and AF2 initial guess for protein–protein complex prediction were insufficient to discriminate successful from unsuccessful binders, even for experimentally active complexes. AF3 showed a clearer separation between the control set and the nonbinding designs but failed to confidently predict the redesigned variants R2–6.1 and R2–6.2. This may reflect the higher binding affinities of the control set (from pM to low nM range), which could make these complexes easier for AF3 to predict accurately than our weaker binders (low μM range). These results highlight a current challenge in accurately predicting designed binders with moderate affinities. Depending on the target, such binders are often the best outcomes of purely computational design campaigns and can nevertheless provide valuable starting points for subsequent optimization and affinity maturation. In contrast, molecular dynamics simulations captured dynamic features of the complexes that static structure prediction could not resolve. In particular, analyses simultaneously quantifying intramolecular flexibility and rigid-body motions of the binder relative to the target clearly separated successful and unsuccessful design classes. Excessive rigid-body fluctuations emerged as a recurrent characteristic of failed interfaces, suggesting that interface dynamics constitute an underappreciated determinant of binder quality.

Spatial aggregation propensity (SAP) emerged as another particularly informative metric. Although SAP has previously been proposed to evaluate aggregation-prone regions, it has been only sparsely adopted in *de novo* protein binder design. Our results show that SAP provides a simple yet powerful means to quantify one of the principal trade-offs governing monomeric binder design. Surface hydrophobicity therefore emerges as a limited design resource that must be carefully distributed rather than simply minimized or maximized. Exposed hydrophobic residues can contribute to strong binding interfaces, but excessive hydrophobicity can promote self-association and oligomerization, whereas insufficient hydrophobicity may limit binding affinity. Consequently, successful binders appear to occupy a relatively narrow window of total interface hydrophobicity while concentrating hydrophobic residues specifically at the binding interface, as illustrated by the control binders. In our data set, total SAP values between approximately 30 and 45 were consistently associated with well-behaved binders, although future studies across additional targets will be required to determine how broadly this range applies. More generally, our results emphasize that exposed hydrophobicity should be regarded as a limited design resource that must be strategically allocated.

One limitation of this study is the nature of the control set. The design models available for this study correspond to binders that had undergone yeast selection followed by affinity maturation, processes that may have introduced subtle sequence changes that are difficult to rationalize but can have substantial effects on binding and/or stability. Consequently, these sequences may be more difficult to obtain directly through computational design, and the distribution of scores observed for the control set may not be representative of those expected for successful binders generated solely through computational design. Despite this limitation, the simulated properties of the control set and their comparison with those of the Round 1 and Round 2 designs proved highly informative for rationalizing the origins of experimental failures and guiding the subsequent design rescue.

Despite being generated with an earlier generation of computational design methods, we believe that the principles identified in this study remain broadly applicable to contemporary AI-driven protein design pipelines. Recent approaches, including RFdiffusion and ProteinMPNN-based workflows, have substantially improved binder design success rates, yet they still address the same underlying challenge: the simultaneous optimization of multiple competing objectives, including folding, binding, and monomeric behavior. Consequently, orthogonal validation using complementary physics-based approaches remains valuable for identifying failure modes that are not explicitly captured by current generative models. For example, we have shown that although SolubleMPNN redesign generally improves the SAP scores of Round 2 designs, a notable fraction of the resulting sequences still exhibit high SAP values, highlighting the need for careful assessment of this property. Our framework complements current approaches by providing mechanistic interpretation of design failures and practical criteria for prioritizing candidates before experimental characterization.

Importantly, our objective was not simply to obtain an improved binder using the latest design technologies, but rather to determine whether the identified failure mechanisms could be corrected through targeted structural modifications. For this reason, we deliberately preserved the original R2–6 backbone and applied only localized redesign guided by the computational analyses instead of generating entirely new backbones with RFdiffusion or redesigning the sequence using ProteinMPNN. By preserving the original backbone, we were able to isolate the effect of the introduced modifications. Properties predicted to be satisfactory, including folding quality and interface architecture, remained unchanged, whereas the predicted cause of failure—excessive surface hydrophobicity—was corrected. This direct correspondence between computational predictions and experimental outcome provides strong evidence that the identified failure mechanisms are causal rather than merely correlative.

The rapid development of AI-based protein design methods is substantially increasing the accessibility and success rate of *de novo* binder design. As these methods continue to evolve, however, understanding the molecular basis of design success and failure will remain essential for improving experimental outcomes. We anticipate that the failure modes, computational assessment framework, and design principles described here will provide practical guidelines for interpreting computational predictions, prioritizing candidate binders, and guiding the rational optimization of existing designs. More broadly, our results suggest that future advances in *de novo* protein binder design will depend not only on increasingly powerful generative algorithms, but also on mechanistic understanding of the trade-offs between folding, binding, and monomeric behavior.

## Methods

### Computational Design of Protein Binders

The crystal structure of the SARS-CoV-2 receptor-binding domain (RBD) in complex with the human ACE2 receptor (PDB ID: 6M0J) served as the structural template for binder design. Protein backbones comprising three- and four-helix bundles were generated to recapitulate interactions between the ACE2 α1 helix and the RBD. Backbone generation was performed using the Blueprint Builder mover in RosettaScripts^24^ through Monte Carlo fragment assembly. Input blueprint files were generated with custom Python scripts. To preserve the native binding geometry, the ACE2 α1 helix (residues S19–N51) was incorporated as a fixed structural motif during backbone construction. To reduce scaffold size while retaining all RBD-contacting residues, between 7 and 12 residues were removed from the C-terminus of α1, yielding fixed helical motifs of 18–24 residues. The remaining helices were built by systematically sampling helix lengths of 18–24 residues connected to one another and to the fixed helical motif by loops of 2–4 residues. Loop geometries were initially specified using ABEGO patterns^25^ that favor N-terminal hydrogen-bonded capping with the following helix (XB, XXB, and XXXB). ABEGO patterns describe local backbone geometries by assigning backbone torsion angles to discrete bins corresponding to distinct regions of the Ramachandran plot: “A” for right-handed α-helix, “B” for extended β-strand, “E” for extended regions with positive ϕ angles, “G” for left-handed α-helix, and “O” for nonplanar peptide bonds. The “X” torsion bin is used to avoid biasing the ABEGO in that particular position during fragment picking. Generated backbones were subsequently filtered to retain only structures with nonkinked helices, high compacity, and favorable loop conformations, prioritizing loops with GB, GBB, BAB, GBBB, AGBB, or ABAB ABEGO types. To promote well-packed helical bundles, harmonic distance constraints were applied between residues from different helices according to the expected parallel or antiparallel helix pairings for each bundle topology. For four-helix scaffolds, an additional geometric filter ensured that the fourth helix remained in close proximity to a helical loop connecting strands β6 and β7 of the RBD (residues 482–491), which natively makes extensive contacts with a β-hairpin of ACE2.

Thousands of filtered backbones were first subjected to sequence design calculations with the FastDesign mover^19^ using the ref 2015 energy function^26^ in the absence of the target to optimize binder folding and monomeric behavior. Residues on α-helices were restricted to incorporate amino acid identities based on their solvent accessibility, and those on connecting loops were designed using consensus sequence profiles derived from fragments with the same ABEGO torsions.^27^ Designs exhibiting low total Rosetta energies (<−3.5 Rosetta Energy Units per residue), well-packed hydrophobic cores, and shape-complementary interhelical packing subsequently underwent a second round of FastDesign in the presence of the RBD to optimize binding interactions while preserving the scaffold architecture. The backbone relaxation implicit in FastDesign led to multiple backbone perturbations with respect to the starting helix motif that favored binding interface contacts. Final designs were ranked based on the Rosetta ddG binding energy, interface shape complementarity, and buried surface area. Next, the local compatibility between the designed backbones and sequences was assessed through fragment quality analysis,^18,28^ and those with the best fragment at the worst residue position with an RMSD of 1.5 Å were selected for Rosetta *ab initio* folding simulations, generating about 5000 trajectories per design, and those with funnel-shaped energy landscapes were considered for experimental testing.

### AlphaFold Predictions

AlphaFold2 (AF2) and AlphaFold2-Multimer (AFM; v2 weights) predictions were performed using ColabFold^29^ (v1.5.3) installed on a local computing cluster. AF2 initial guess predictions were run by initializing the pair representation of the AF2 network using the design model structures as described previously.^1^ AF3 (v3.0.0) predictions were performed locally. All AF2 monomeric predictions were run in single-sequence mode using three recycling iterations. For protein–protein complex predictions with AFM and AF3, MSA searches were enabled and the target receptor structure was provided as a template. Predicted structures were subsequently relaxed with the Amber force field. The top-ranked AFM predicted model was used for structural and confidence analysis. AF3 predictions were run with 100 seeds (with 5 diffusion samples each) and the top-ranked model based on ipTM was used for analysis. For each prediction, structural agreement with the design model was assessed using Cα-RMSD and TM-score. Prediction confidence was evaluated using pLDDT, pTM, and PAE; ipTM was additionally used for AFM and AF3 complex predictions. For complex predictions, binder Cα-RMSD (L-RMSD) was calculated after superimposing the predicted and design complexes using the target receptor as the alignment reference.

### Molecular Dynamics Simulations

Molecular dynamics (MD) simulations were performed using GROMACS 2021.4 ^30^ with the Amber ff99SB-ILDN force field.^31^ Each protein system was solvated in a dodecahedral periodic box containing TIP3P water molecules, maintaining a minimum distance of 11 Å between the protein and the box boundaries. Na^+^ and Cl^–^ ions were added to neutralize the system and achieve a final ionic concentration of 0.137 M. Energy minimization was carried out for 50,000 steps using the steepest descent algorithm. The systems were then heated from 100 to 300 K over 500 ps, followed by equilibration for 500 ps in the canonical (NVT) ensemble at 300 K and an additional 1 ns in the isothermal–isobaric (NPT) ensemble at 300 K and 1 bar. Long-range electrostatic interactions were calculated using the particle mesh Ewald (PME) method, while short-range Lennard–Jones and Coulomb interactions were evaluated with a 10 Å cutoff. Covalent bonds involving hydrogen atoms were constrained using the LINCS algorithm, enabling a 2 fs integration time step. For each protein complex, three independent 100 ns production simulations were performed, with trajectory coordinates saved every 50 ps. Trajectory analysis was conducted using GROMACS utilities and custom Python scripts based on the MDAnalysis library.^32^ To ensure that only equilibrated conformations were analyzed, the initial 30 ns of each production trajectory were excluded from subsequent analyses.

### Spatial Aggregation Propensity (SAP) Analysis

To estimate the propensity of designed binders to self-associate or aggregate, we computed the Spatial Aggregation Propensity (SAP)^22^ using the Rosetta implementation.^6^ SAP quantifies solvent-exposed hydrophobic patches, with higher scores indicating larger, exposed aggregation-prone regions, whereas lower scores correspond to hydrophilic or buried protein surfaces. Per-residue SAP scores were calculated for all designs and summed to obtain the total SAP score. To distinguish aggregation propensity associated with the binding interface from that of the remaining protein surface, the total SAP score was further decomposed into contributions from interface and noninterface residues. Interface residues were identified based on the InterfaceByVector residue selector in Rosetta.

### LightDock Docking Calculations

Protein–protein docking simulations were performed using LightDock^21^ (v0.9.3), which applies a swarm-based docking algorithm to sample multiple orientations and conformations of interacting partners. The Membranator tool from the LightDock server^33^ was used to generate a “false” plain membrane model around the receptor, for which a biologically relevant anchor residue was manually selected based on preliminary structural information. This allowed the docking search space to be restricted to the expected binding interface while preventing sampling of irrelevant regions. Docking was initialized using 76 swarms in the extramembranous region of the membrane with 200 glowworms each. After docking, the resulting poses were ranked using the DFIRE^34^ scoring function (the default scoring metric in LightDock), subsequently clustered (to reduce very similar and redundant models per swarm) and the top-100 LightDock predictions were selected. The final top-10 docked complexes were refined using Rosetta FastRelax^5^ and assessed for docking accuracy using DockQ. Models were classified under the Critical Assessment of PRediction of Interactions (CAPRI) criteria, which assigns the model into one of four discrete accuracy classes based on the model’s similarity to the native structure based on DockQ ranges: incorrect (DockQ < 0.23), acceptable (0.23 ≤ DockQ < 0.49), medium (0.49 ≤ DockQ < 0.80), and high (DockQ ≥ 0.80).

### Protein Expression and Purification

The designed constructs were engineered to contain an N- or C-terminal 6 × His tag followed by a Tobacco Etch Virus (TEV) protease cleavage site. The constructs were cloned into the pET-24a­(+), pET-29­(+) or pET-28a­(+) expression vectors, and the final plasmids were purchased from Genscript (see Table S1 for final construct sequences). The constructs were expressed in *E. coli* BL21 (DE3) strain (Invitrogen). Transformed cells were grown in LB medium supplemented with 50 μg mL^–1^ kanamycin at 37 °C until an optical density at 600 nm (OD600) of 0.6 was reached. Expression was induced with 100 μM isopropyl-ß-d-thiogalactopyranoside (IPTG) for 16 h at 16 °C. Cells were pelleted by centrifugation at 4500*g* for 30 min and then resuspended in 40 mL of phosphate-buffered saline (PBS 1×). Finally, cells were harvested by centrifugation and stored at −80 °C. For protein purification, cells were resuspended in PBS 1× buffer supplemented with 1 complete EDTA-free protease inhibitor (Roche) tablet, 0.5 mg mL^–1^ lysozyme, 10 μg·mL^–1^ RNaseA and 10 μg·mL^–1^ DNase and lysed by sonication. Cellular debris were pelleted by centrifugation. The soluble fraction was incubated 1 h at 4 °C with 1 mL of pre-equilibrated TALON resin (TAKARA). After three washes with 5 mL of PBS 1× supplemented with 5 mM of Imidazole to remove unbound proteins, the constructs were eluted with 2 mL of PBS 1× containing 500 mM imidazole. Finally, the eluate containing the constructs was further purified by size exclusion on a Superdex 75 10/300 (GE Healthcare) in PBS 1×. Protein expression and purity were assessed by SDS-PAGE, and proteins were frozen in liquid nitrogen and stored at −80 °C. The expression of purified proteins was assessed by SDS-polyacrylamide gel; and protein concentrations were determined from the absorbance at 280 nm measured on a NanoDrop spectrophotometer (ThermoScientific) with extinction coefficients predicted from the amino acid sequences using the ProtParam tool.

### SEC-MALS

Size-exclusion chromatography coupled to multiangle light scattering (SEC-MALS) was performed using a DAWN HELEOS II system (Wyatt Technologies) connected to a Superdex 75 Increase 10/300 GL column (Cytiva). The column was equilibrated with PBS and operated at 25 °C with a flow rate of 0.5 mL/min. Eluting samples were monitored simultaneously by UV absorbance (SPD-20, Shimadzu), differential refractive index (Optilab rEX, Wyatt Technologies), and multiangle light scattering (DAWN HELEOS II, Wyatt Technologies). Data acquisition and molecular weight determination were performed using ASTRA 7 software (Wyatt Technologies).

### Circular Dichroism

Far-UV circular dichroism (CD) measurements were performed on a JASCO J-1500 spectropolarimeter at the IRB/IBMB Automated Crystallography Platform to assess protein secondary structure and thermal stability. Protein samples were prepared in PBS buffer (pH 7.4) at a concentration of 0.3 mg/mL and analyzed with a 1 mm path-length quartz cuvette. Wavelength scans were recorded from 260 to 190 nm at 20, 30, 40, 50, 60, 70, 80, 90, 95 °C and again at 25 °C after refolding. Temperature melts monitored the measured ellipticity at 220 nm.

### Surface Plasmon Resonance

Surface plasmon resonance (SPR) measurements were performed on a Biacore T200 instrument (Cytiva). Biotinylated SARS-CoV-2 (2019-nCoV) receptor-binding domain (RBD; Sino Biological, Cat. No. 40592-V08B–B) was immobilized onto a Series S streptavidin sensor chip (Cytiva) in HEPES running buffer containing 0.05% (v/v) Tween-20 at 25 °C. Approximately 400 response units (RU) of RBD were captured on channel 2, while channel 1 was kept as a reference. Analyte binders were injected at six concentrations (0.1, 0.5, 2, 6.25, 12.5, and 25 μM), with all measurements performed in duplicate and in order of increasing concentration. Blank buffer injections were included between sample injections for double referencing and baseline correction. Association and dissociation phases were monitored for 60 and 300 s, respectively, at a flow rate of 30 μL/min. Sensorgrams were processed using Biacore Evaluation Software (Cytiva) and fitted with a 1:1 Langmuir binding model to determine binding kinetics (*k*_on_ and *k*_off_) and equilibrium dissociation constants (*K*_D_). For R2–6.1 and R2–6.2, the goodness of the fit was verified by confirming that the χ^2^ value was less than 10% of the maximum response (*R*_max_).

### ELISA Binding Assays

High-binding 96-well microplates (Nunc MaxiSorp) were coated overnight at 4 °C with 50 μL per well of recombinant SARS-CoV-2 (2019-nCoV) receptor-binding domain (RBD) fused to human Fc (Sino Biological, Cat. No. 40592-V02H) at 2 μg/mL in PBS. Wells were blocked with 2% (w/v) BSA IgG-Free (Jackson) in PBS for 2 h at 37 °C and washed four times with PBS. Serial dilutions of recombinant His-tagged binders and human ACE2 (Sino Biological, Cat. No. 10108-H08B) prepared in PBS containing 1% BSA were added to the wells and incubated for 1 h at 37 °C. After three washing steps, bound proteins were detected using an HRP-conjugated mouse anti-His antibody (Sino Biological, Cat. No. 105327-MM02T1H) for 1 h at 37 °C. Plates were washed four additional times before color development with *o*-phenylenediamine (OPD) substrate prepared in phosphate-citrate buffer immediately before use by addition of hydrogen peroxide. The reaction was stopped with an equal volume of 2.5 M sulfuric acid, and absorbance was measured at 490 nm using a BioTek PowerWave XS microplate reader.

### Molecular Visualization and Graphical Representation

Molecular graphics images were produced using PyMOL.^35^ Data analyses were performed with numpy, seaborn and matplotlib.^36^

## Supplementary Material



## Data Availability

The designed protein sequences are provided in Table S1. Design and predicted models, simulated data, execution and analysis scripts have been deposited in the Zenodo database (https://zenodo.org/records/23268880). The Rosetta macromolecular modeling suite (http://www.rosettacommons.org) is freely available to academic and noncommercial users.

## Notes

Simulated folding energy landscapes of the Round 1 (Figure S1) and Round 2 (Figure S2) designs; Size-exclusion chromatography analysis of Round 1 designs (Figure S3); ELISA binding assays (Figure S4); Surface plasmon resonance (SPR) sensorgrams for selected Round 1 designs (Figure S5); Size-exclusion chromatography combined with multiangle scattering data for Round 2 designs (Figure S6); AlphaFold evaluation of binder-target complexes using AlphaFold2 initial guess and AlphaFold3 (Figure S7); Molecular dynamics simulations of the control (Figure S8), Round 1 (Figure S9) and Round 2 (Figure S10) designs; Spatial aggregation propensity (SAP) scores normalized by sequence length (Figure S11); SAP scores for SolubleMPNN redesigns (Figure S12); Designed amino acid sequences of the experimentally characterized proteins (Table S1); Size-exclusion chromatography combined with multiangle scattering data for the rescued designs (Table S2) (PDF)
