# Supplementary material for: Design Trade-Offs between Target Binding and Self-Association Revealed by De Novo SARS-CoV‑2 Binders
Source: J Chem Inf Model. 2026 Oct 27;66(21):14053–67. doi: 10.1021/acs.jcim.6c02394 (PMC13753715; doi:10.1021/acs.jcim.6c02394)
Supplement: Supplementary file 1 [file ci6c02394_si_001.pdf]

# Supporting Information

# Design Trade-Offs between Target Binding and Self-Association Revealed by De Novo SARS-CoV-2 Binders

Lourdes Carcelén[1], Daniel Peñas-Utrilla[1], Diego S. Ferrero[2], Cristina Ferrer-Orta[2], Marta Nadal[1], Francesco Colizzi[3,4], Juana C. Muñoz[1], Marta Taulés[5], Núria Verdaguer[2], Enrique Marcos[1*]

[1]Protein Design and Modeling Lab, Department of Structural Biology, Molecular Biology Institute of Barcelona (IBMB), CSIC, Baldiri Reixac 10, 08028 Barcelona, Spain

[2]Virus and Large Biological Complexes, Department of Structural Biology, Molecular Biology Institute of Barcelona (IBMB), CSIC, Baldiri Reixac 15, 08028 Barcelona, Spain

[3]Molecular Ocean Lab, Institute for Advanced Chemistry of Catalonia (IQAC), CSIC, Jordi Girona 18-26, 08034 Barcelona, Spain

[4]Department of Marine Biology and Oceanography, Institut de Ciències del Mar (ICM), CSIC, Passeig Marítim de la Barceloneta 37-49, 08003 Barcelona, Spain

[5]Molecular Interactions Analysis Unit, Centres Científics i Tecnològics (CCIT), Universitat de Barcelona (UB), Barcelona, 08028, Spain

*Corresponding author: embcri@ibmb.csic.es

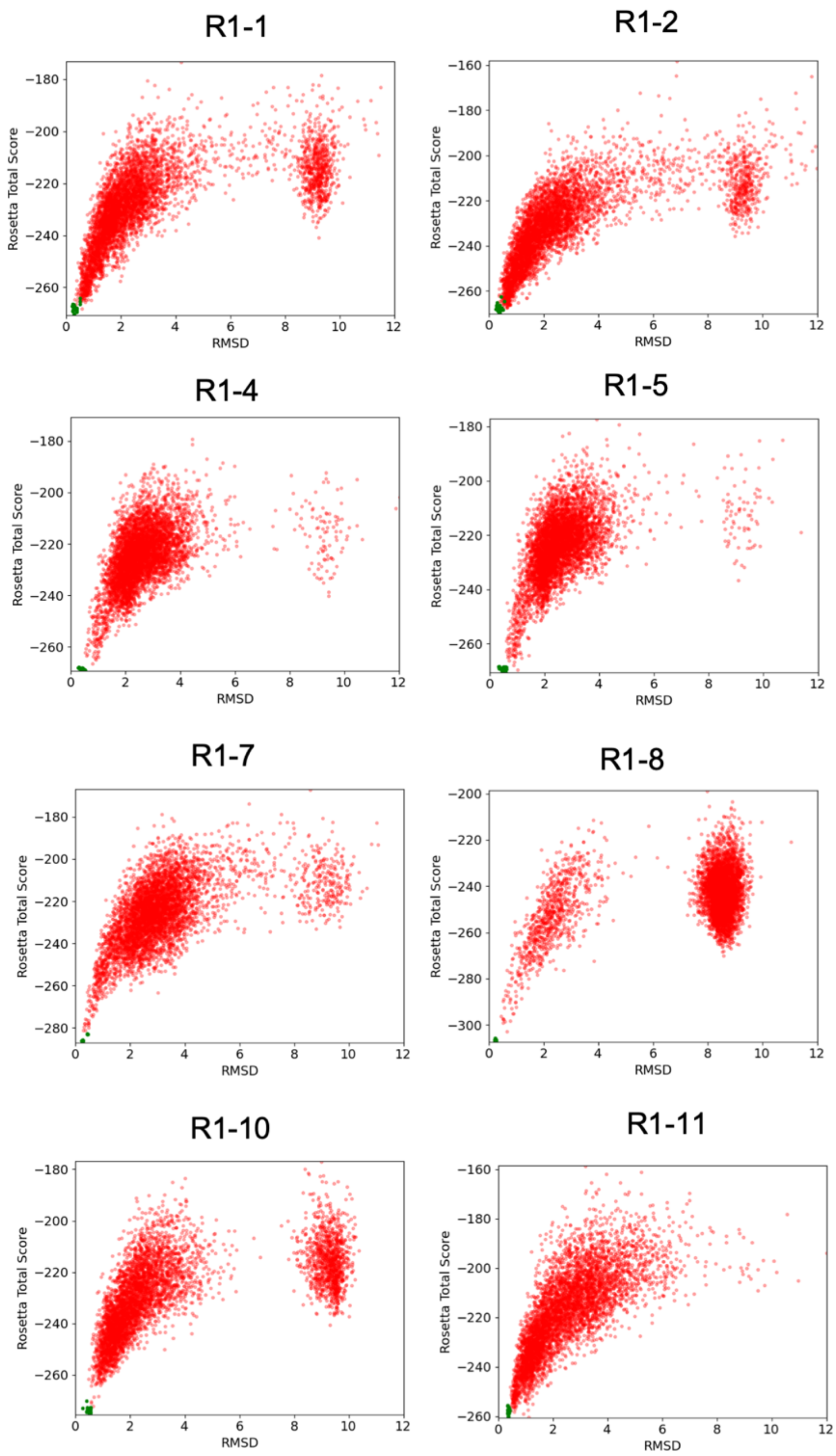


**Figure S1. Simulated folding energy landscapes of the Round 1 designs.** Each dot represents the lowest energy structure obtained from Rosetta ab initio folding trajectories of the monomeric binder starting from an extended chain (red dots) or local relaxation of the designed structure (green dots). The x-axis depicts the Cα-RMSD from the designed model and the y-axis, the Rosetta all-atom energy.

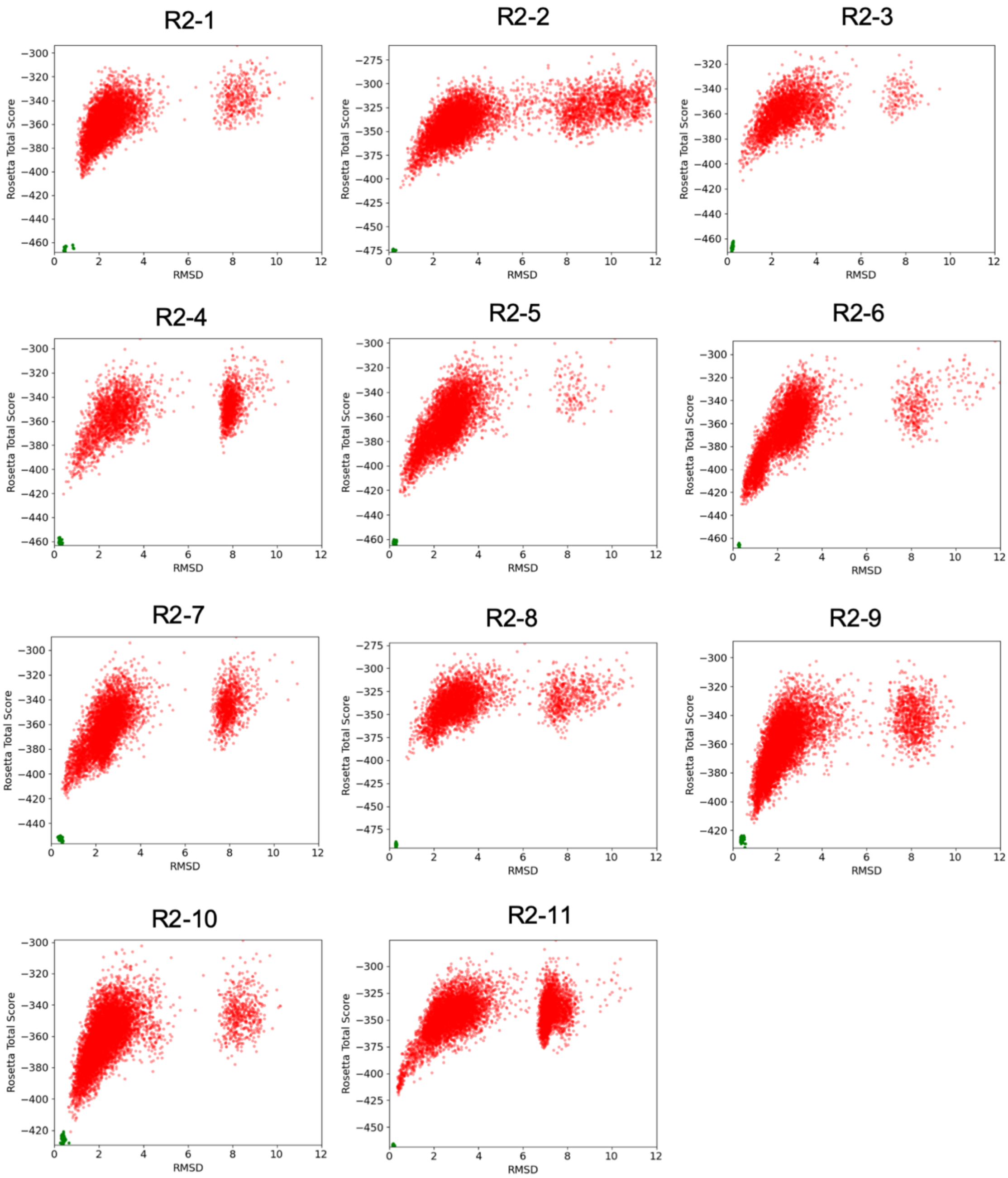


**Figure S2. Simulated folding energy landscapes of the Round 2 designs.** Each dot represents the lowest energy structure obtained from Rosetta ab initio folding trajectories of the monomeric binder starting from an extended chain (red dots) or local relaxation of the designed structure (green dots). The x-axis depicts the Cα-RMSD from the designed model and the y-axis, the Rosetta all-atom energy.

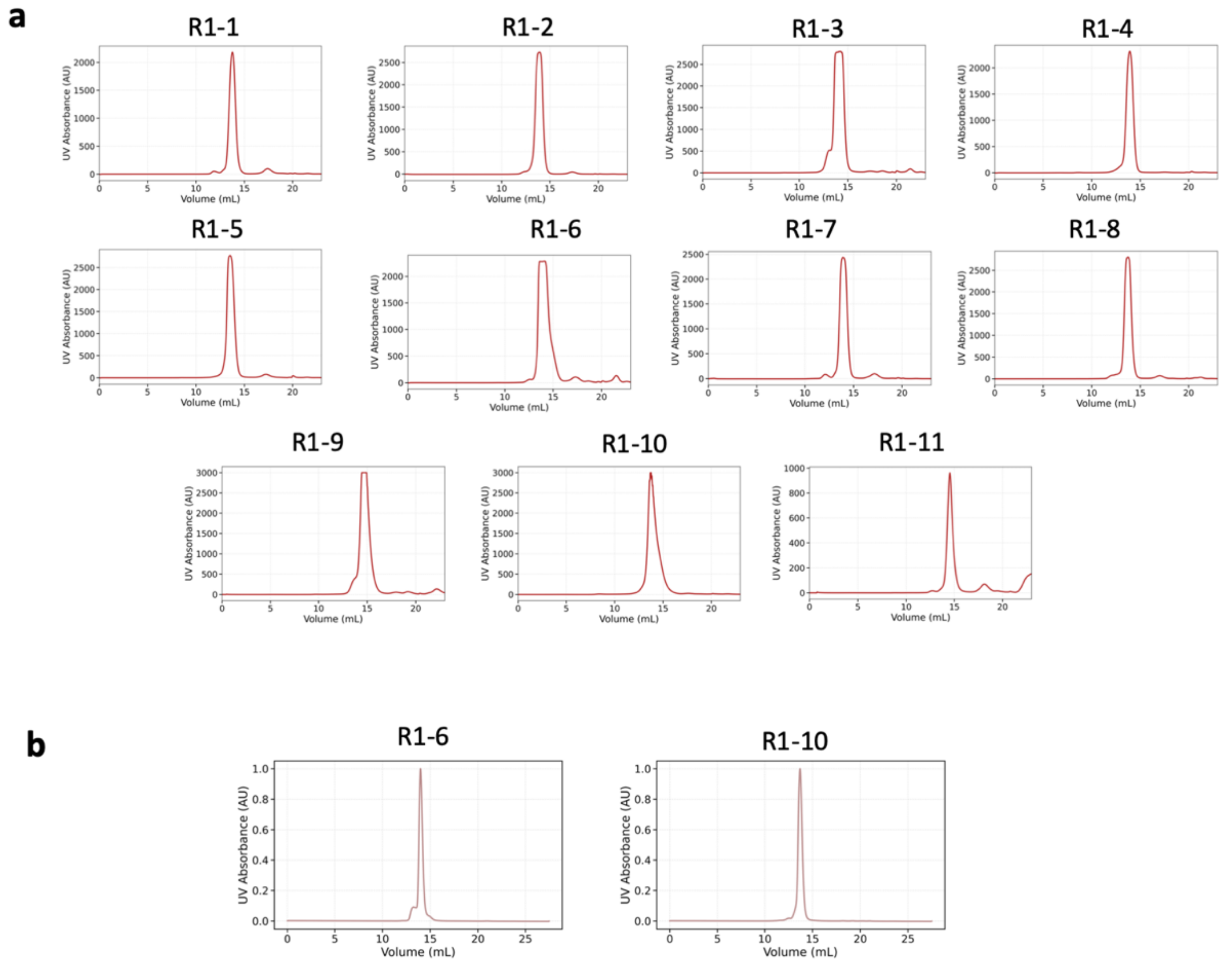


| Design name | Theoretical $M_w$ (kDa) | Estimated Mw (kDa) |
|---|---|---|
| R1-6 | 11.65 | 11.16 (±0.7%) |
| R1-10 | 11.33 | 12.50 (±0.6%) |

**Figure S3. Size-exclusion chromatography analysis of Round 1 designs. (a)** SEC elution profiles of purified Round 1 designs show predominantly monodisperse species, with the major peak eluting at the expected volume based on the monomer molecular weight. **(b)** Representative designs were further characterized by SEC-MALS to determine the oligomeric state of the main elution peak. The estimated molecular weights (Mw) are summarized in the accompanying table and are consistent with monomeric species. The y-axis represents the normalized UV absorbance at 280 nm. All samples were analyzed on a Superdex 75 Increase 10/300 GL column in PBS.

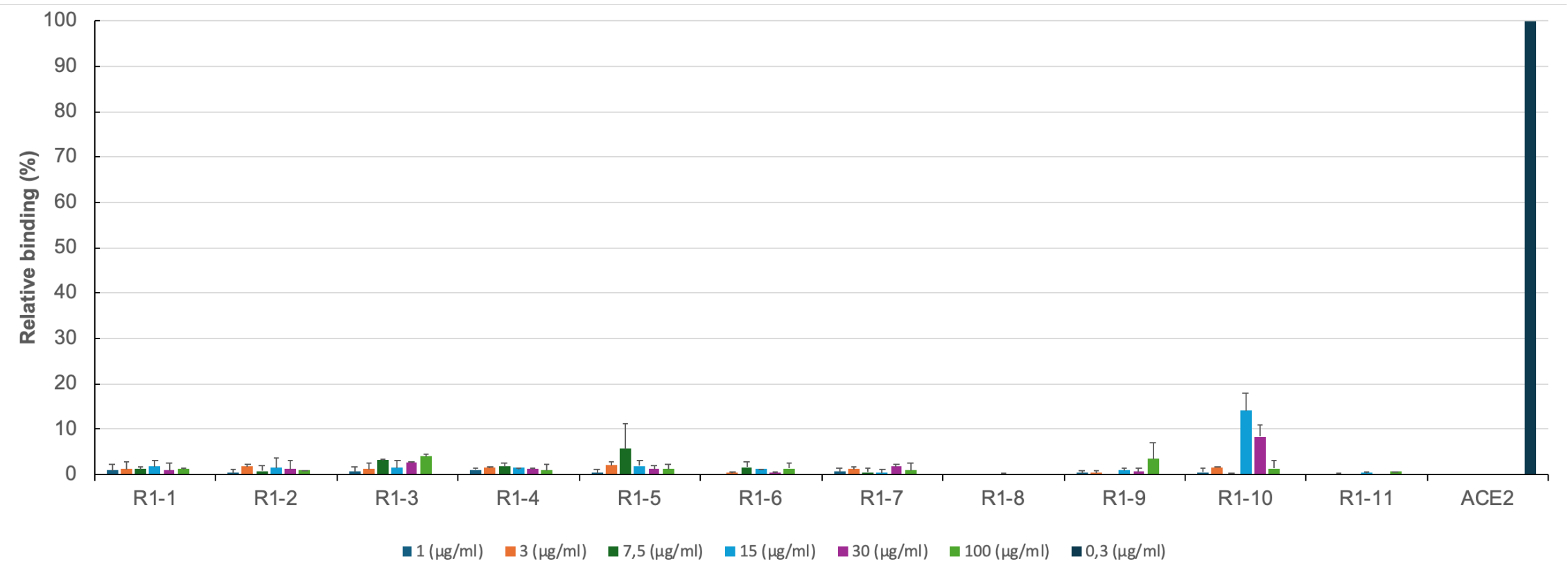


**Figure S4. ELISA-based binding of recombinant His-tagged binders to immobilized SARS-CoV-2 RBD.** Recombinant SARS-CoV-2 receptor-binding domain (RBD)-Fc was immobilized on high-binding microplates and incubated with serial dilutions (0.3–100 µg/mL) of recombinant His-tagged binders (R1-1 to R1-11) or recombinant human ACE2 as a positive control. Bound proteins were detected using an HRP-conjugated anti-His antibody, followed by OPD colorimetric development and absorbance measurement at 490 nm. Binding signals are shown as relative binding (%) normalized to the highest signal obtained with ACE2 (set to 100%). Data are presented as mean ± SD. None of the tested binders exhibited consistent concentration-dependent binding to the immobilized RBD. For ACE2, only the signal at the lowest tested concentration (0.3 µg/mL) is shown because its binding response was substantially higher than that of the tested binders.

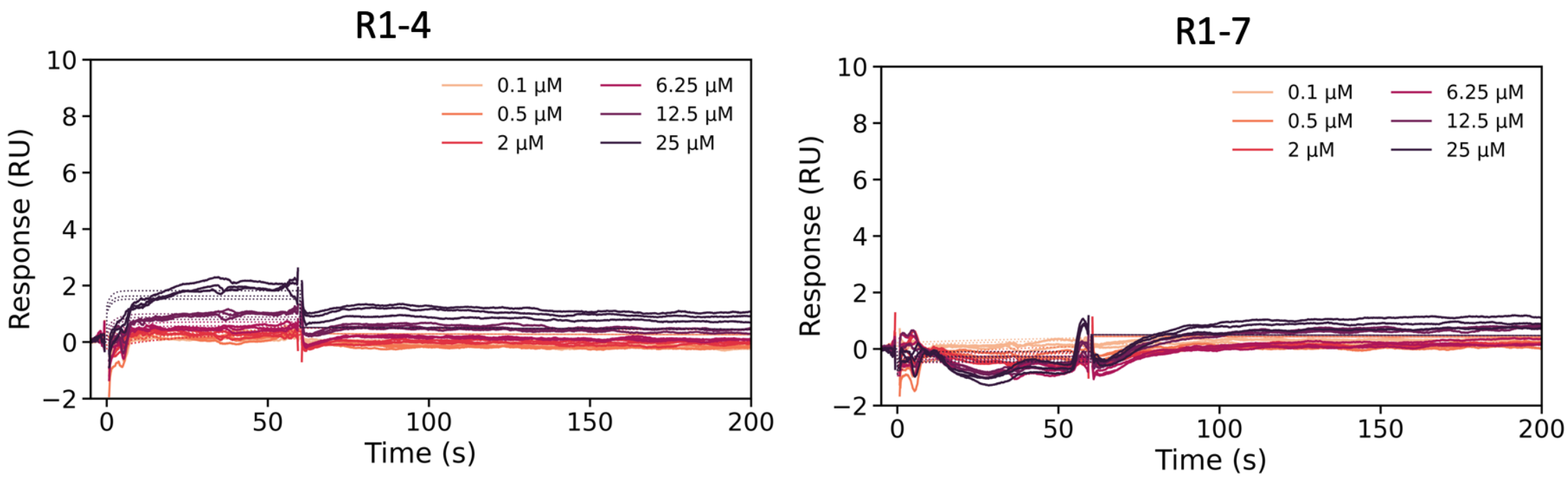


**Figure S5. Representative surface plasmon resonance (SPR) sensorgrams for selected Round 1 designs.** The sensorgrams display weak, noisy responses with no reproducible concentration-dependent binding, indicating that any interaction with the target is below the reliable detection limit under the assay conditions. As a result, kinetic fitting and estimation of the equilibrium dissociation constant were not feasible.

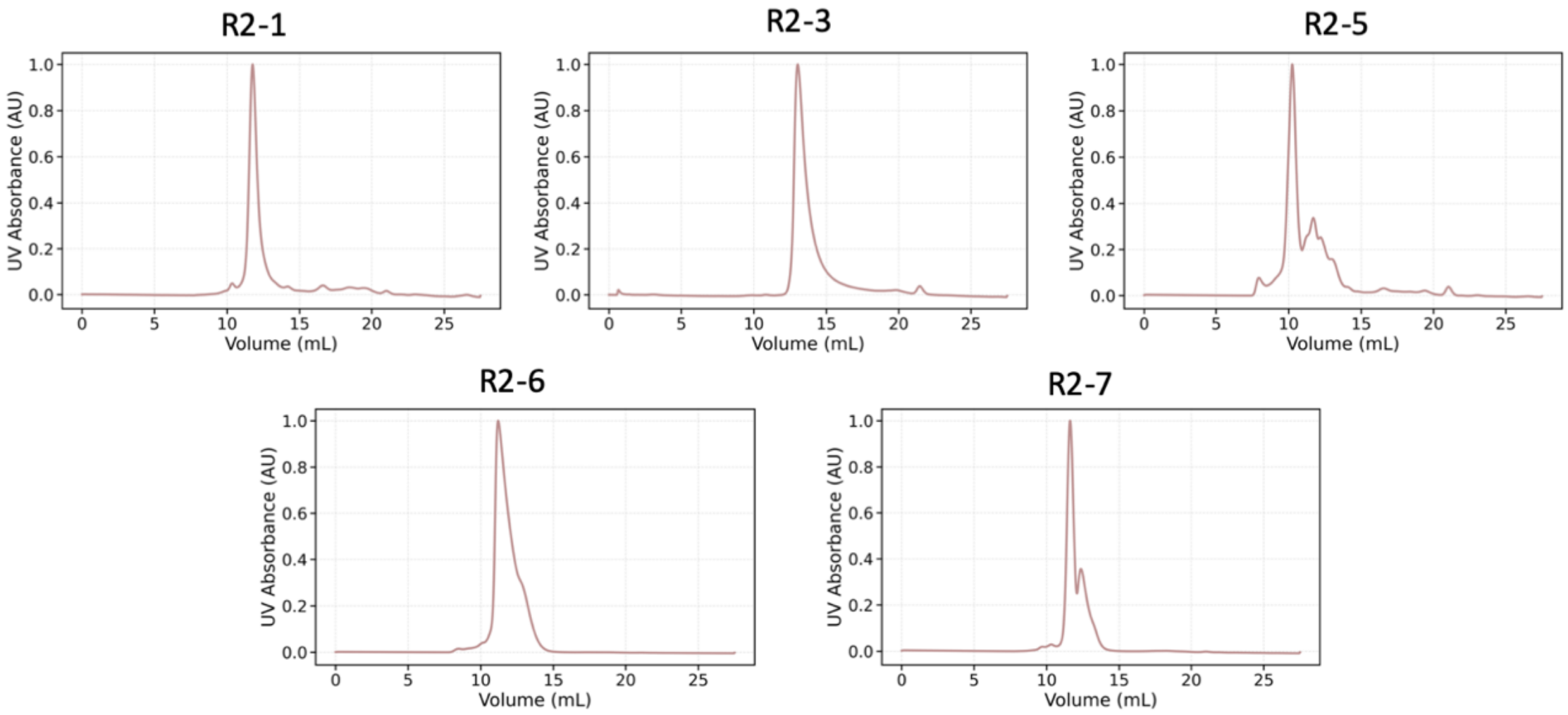


| Design name | Theoretical $M_w$ (kDa) | Estimated Mw (kDa) |
|---|---|---|
| R2-1 | 15.1 | 29.4 (±1.2%) |
| R2-3 | 14.7 | 27.3 (±1.4%) |
| R2-5 | 14.8 | 55.5 (±1.4%) |
| R2-6 | 14.8 | 31.7 (±1.1%) |
| R2-7 | 14.9 | 30.0 (±1.2%) |

**Figure S6. Size-exclusion chromatography combined with multi-angle scattering data for Round 2 designs.** Represented designs correspond to those with soluble expression from Round 2. The y-axis displays the normalized UV absorbance at 280 nm. The table provides the molecular weight ($M_w$) estimated from the main peak of each chromatogram. Most designs (R2-1, -3, -6 and -7) are predicted to be dimers in solution, except for R2-5, which is polydisperse and its main peak corresponds to a tetramer. Samples were run on a Superdex 75 10/300 increase and were prepared in PBS buffer.

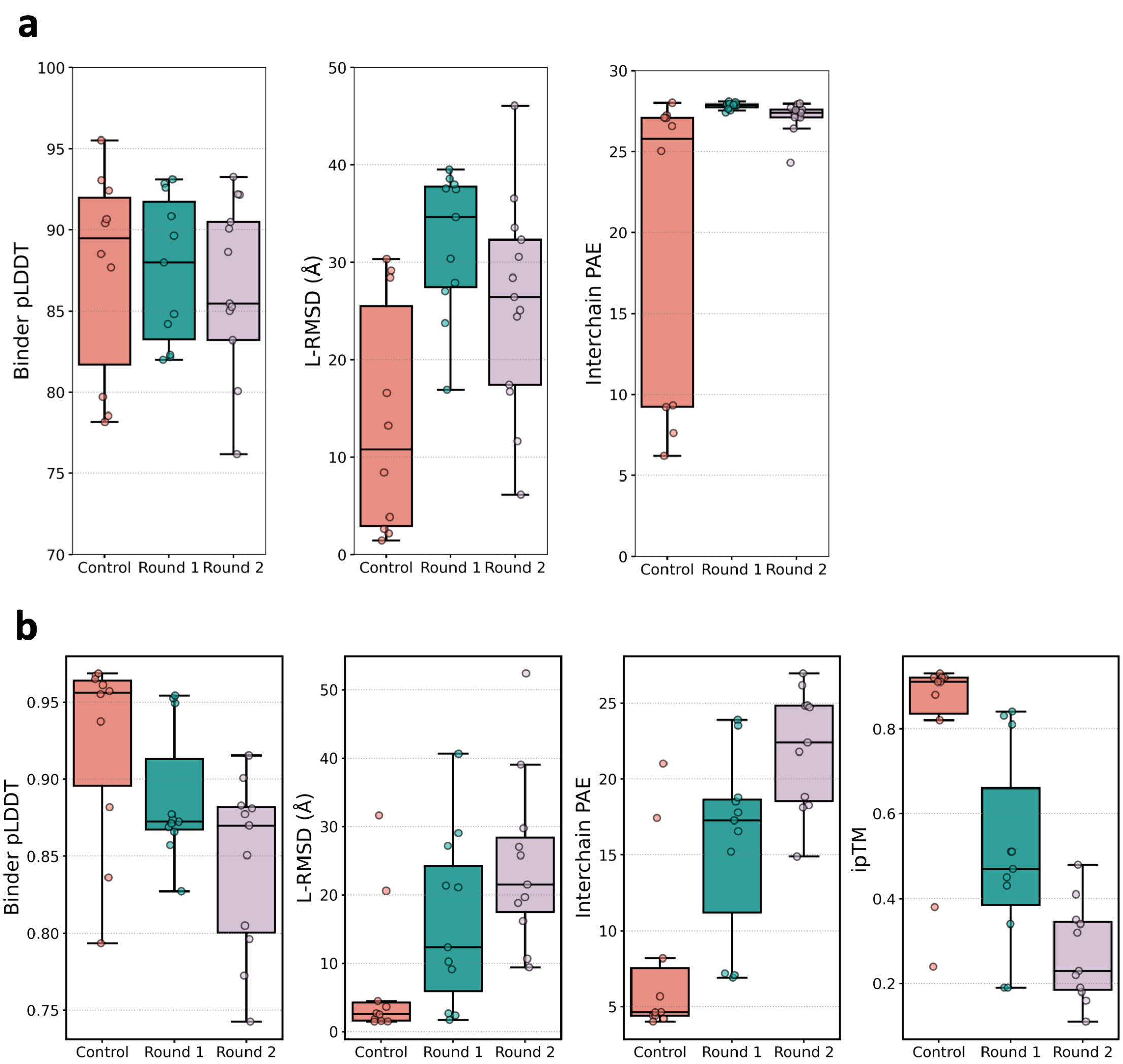


**Figure S7. AlphaFold evaluation of binder-target complexes using AlphaFold2 initial guess (a) and AlphaFold3 (b).** Distributions of binder pLDDT, ligand RMSD (L-RMSD), interchain predicted aligned error (PAE), and ipTM are shown for each design set. AlphaFold3 predictions were performed using 100 seeds and metrics were obtained for the top-ipTM model.

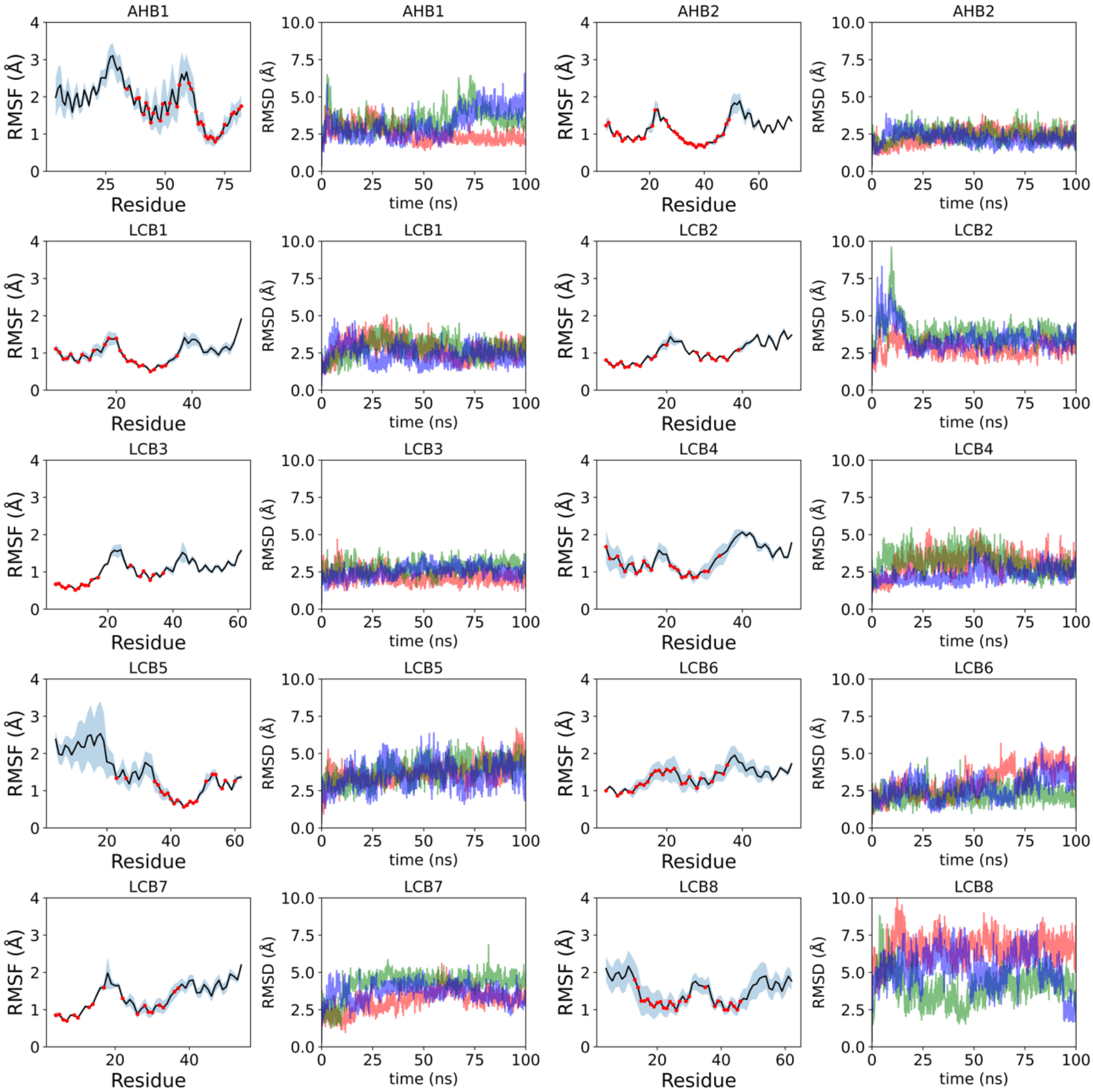


**Figure S8. Molecular dynamics simulations of the control designs.** For each design, the left panel shows the time evolution of the ligand RMSD (L-RMSD) over three independent 100-ns replicas, displayed in different colors. The right panel shows the residue-wise RMSF profile, with red dots indicating binder interface residues. Shaded regions represent the variability among replicas.

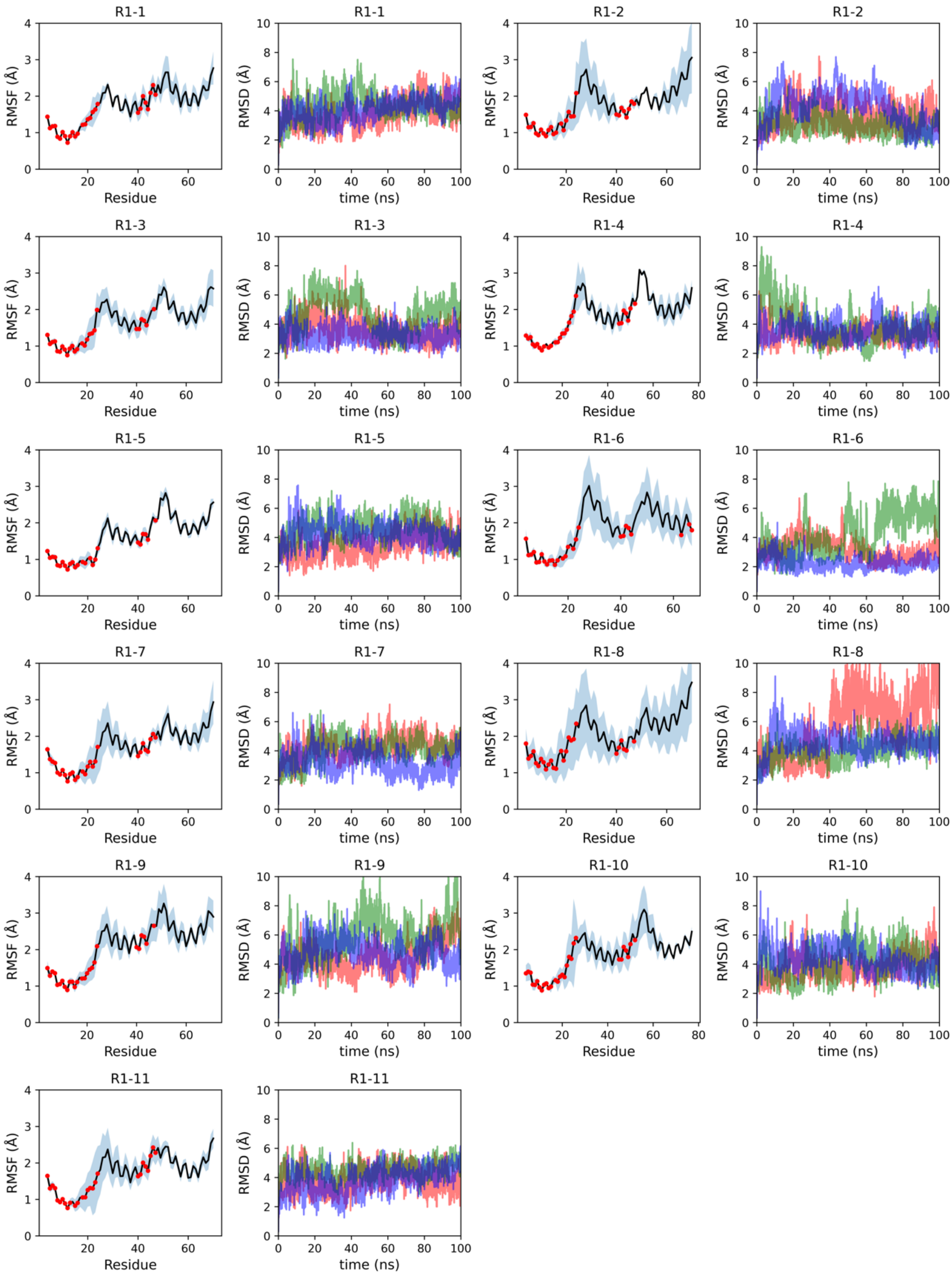


**Figure S9. Molecular dynamics simulations of the Round 1 designs.** For each design, the left panel shows the time evolution of the ligand RMSD (L-RMSD) over three independent 100-ns replicas, displayed in different colors. The right panel shows the residue-wise RMSF profile, with red dots indicating binder interface residues. Shaded regions represent the variability among replicas.

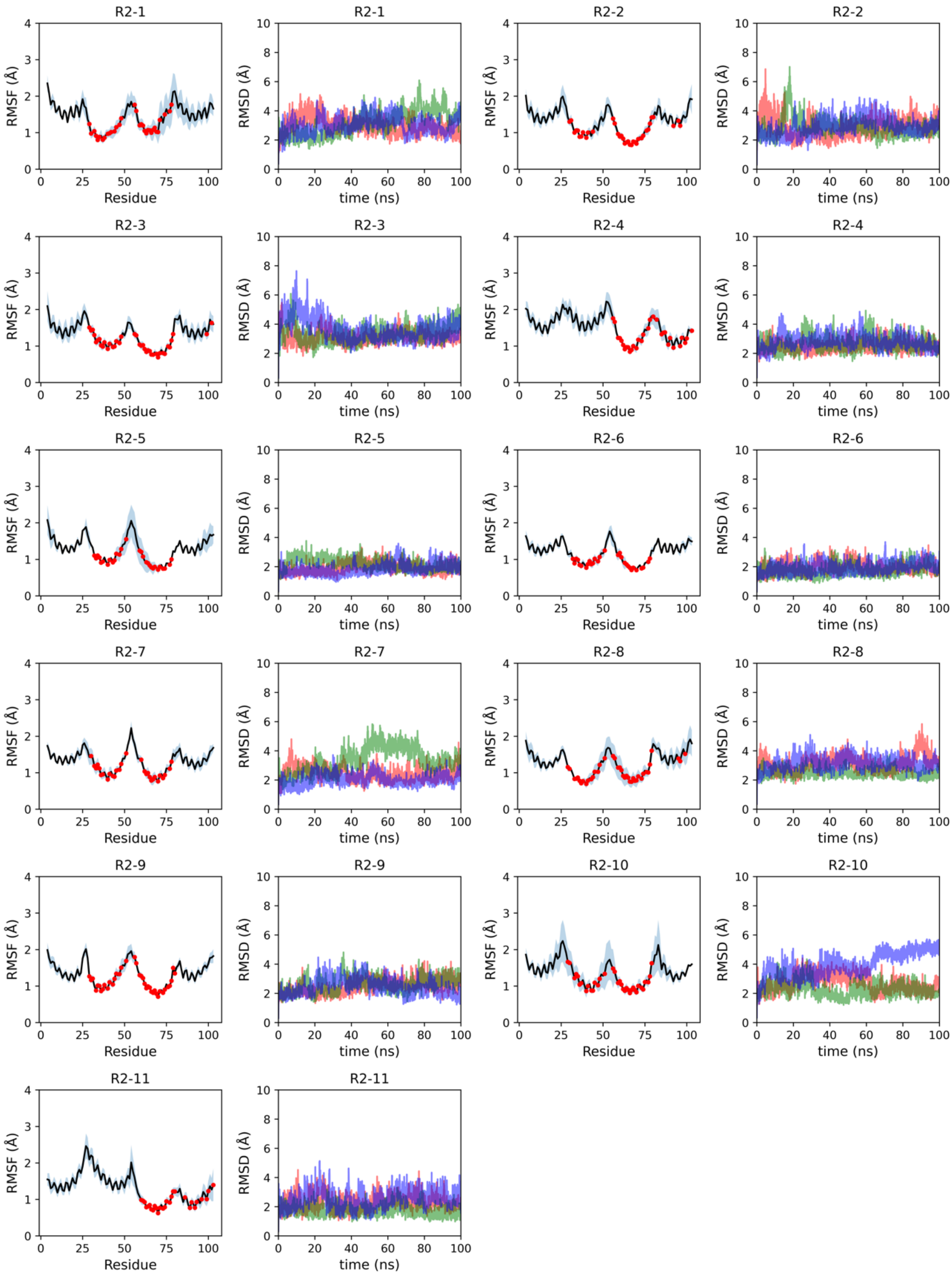


**Figure S10. Molecular dynamics simulations of the Round 2 designs.** For each design, the left panel shows the time evolution of the ligand RMSD (L-RMSD) over three independent 100-ns replicas, displayed in different colors. The right panel shows the residue-wise RMSF profile, with red dots indicating binder interface residues. Shaded regions represent the variability among replicas.

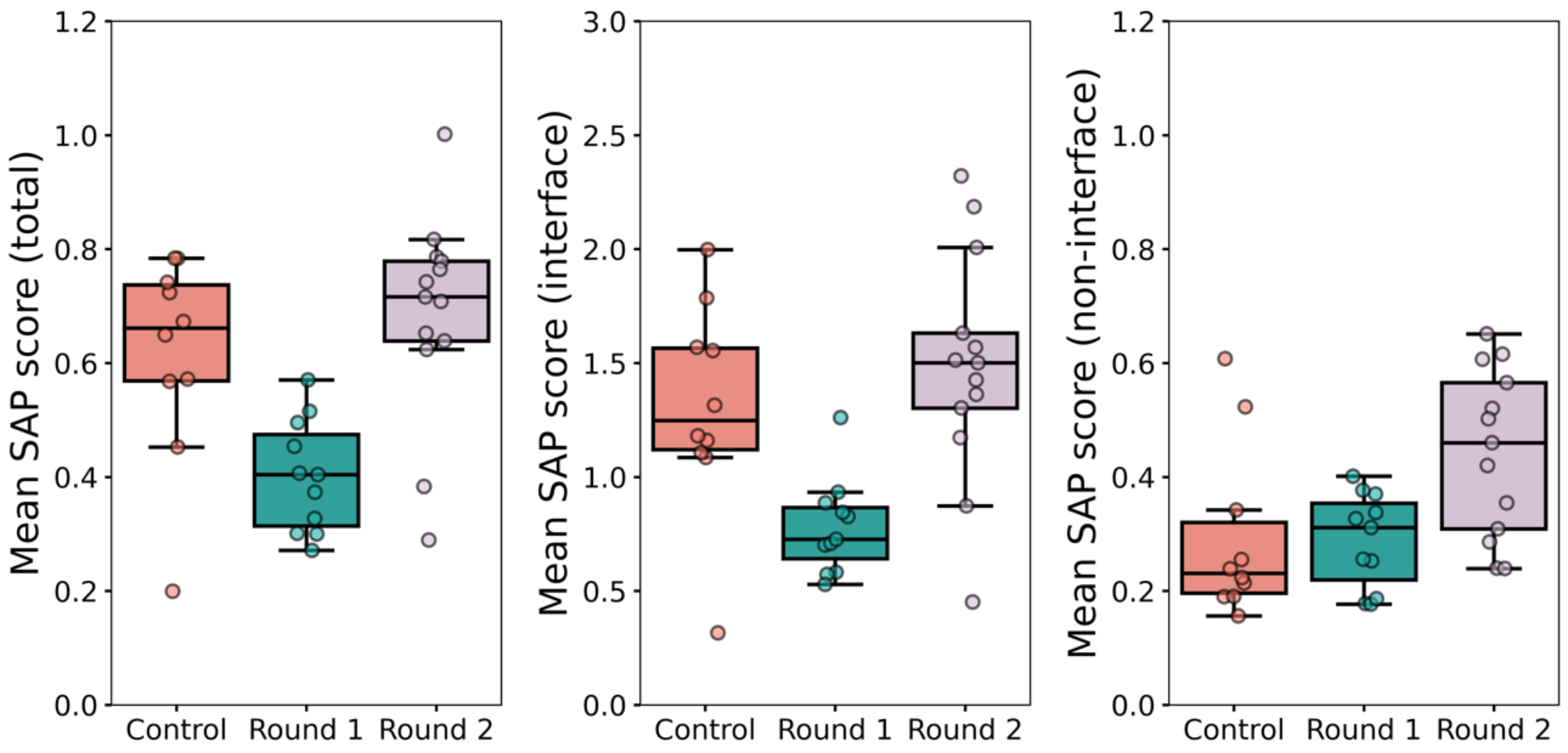


**Figure S11. Spatial aggregation propensity (SAP) scores normalized by sequence length.** Comparison of normalized SAP scores for the control, Round 1, and Round 2 binder sets (left). Although normalization reduces the difference in total SAP between the control set and Round 2 designs compared with the unnormalized analysis shown in Fig. 3, the same overall trends are preserved. Decomposition of the normalized SAP into contributions from interface (middle) and non-interface residues (right) shows that Round 2 designs retain disproportionately high SAP outside the binding interface, indicating that their poor solution behavior is primarily associated with excessive non-interface surface hydrophobicity.

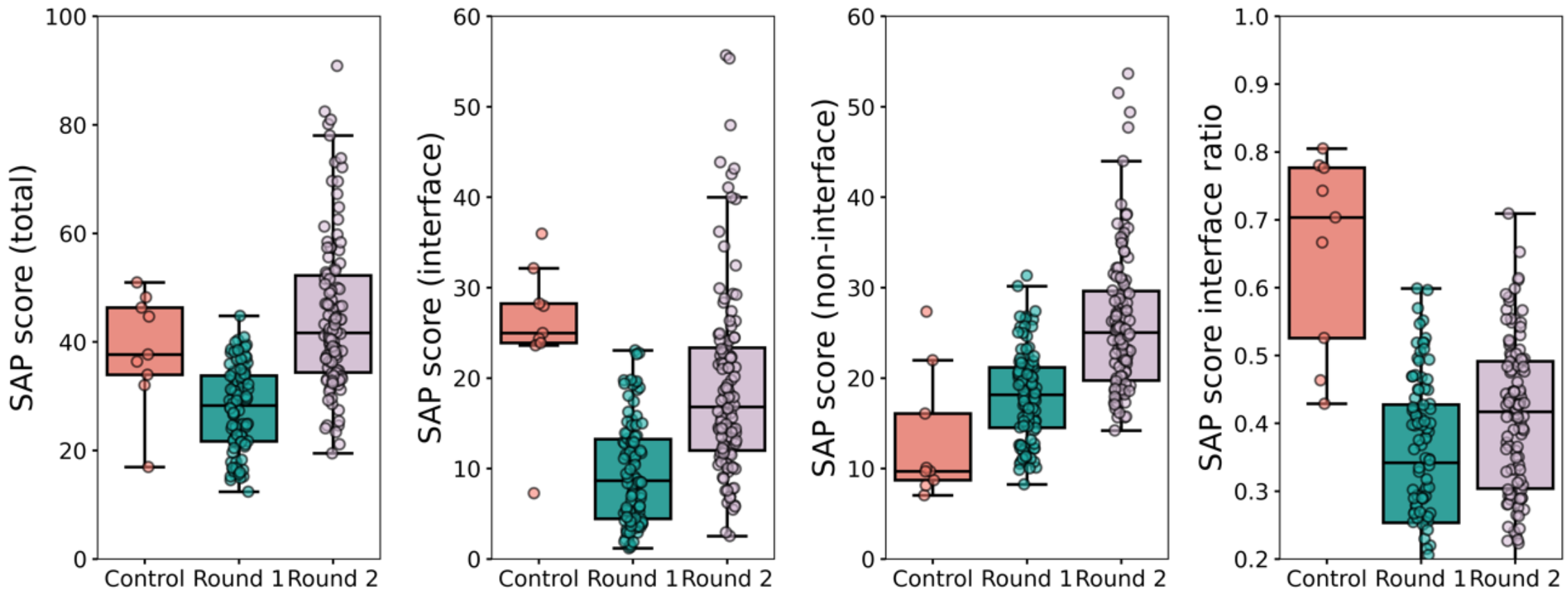


**Figure S12. Spatial aggregation propensity (SAP) scores for SolubleMPNN redesigns.** Comparison of SAP score distributions for the control designs and full-sequence redesigns of the original Round 1 and Round 2 backbones using SolubleMPNN. For each backbone, 10 amino acid sequences were designed.

**Table S1. Designed amino acid sequences of the experimentally characterized proteins.** As indicated in brackets, the N- or C-termini contains a polyhistidine tag and a TEV protease cleavage site.

| Design name | Amino acid sequence |
|---|---|
| R1-1 | MSVEEQAKTFIEKVRHEWEQMEAAGVDPSEMKKALQEWKKLVEMY KEHGLDPSILEEMRKMLEKFEQELKEKES[ENLYFQGLEHHHHHH] |
| R1-2 | MSLEEQAKYFIEKVRHEWEQMEAAGVDPSEMKKALQEWKKWVEMY KEQGLDPSILEEMRKMLEKFEQELKEKES[ENLYFQGLEHHHHHH] |
| R1-3 | MSLEEQAKTFIEKVRHEWEQMEAAGVDPSEMKKALQEWKKYVEMY KEQGLDPSILEEMRKMLEKFEQELKEKES[ENLYFQGLEHHHHHH] |
| R1-4 | MSAEEQLKTFEEKVKHEWEQLKAQNVDPEQLKKIIQEWKKLLQMYE QHGADPEVLKRIKEILKEYEKEAKKLES[ENLYFQGLEHHHHHH] |
| R1-5 | MSAEEQLKLFEEKVKHEWEQLKAQNVDPEQLKKIIQEWKKILQMYEQ HGADPEVLKRIKEILKEYEKEAKKLES[ENLYFQGLEHHHHHH] |
| R1-6 | MSLEEQLKTFWEKFRHEWEQLQQQNASKEELKRWLEKMEELLKMYE KKIQDPRLREQVREYIKSVRKLIEKLEKS[ENLYFQGLEHHHHHH] |
| R1-7 | MSLEEQLKLFWEKFRHEWETLQQQNASKEELKRWLEKMEELLKMYE KKIQDPRLREQVREYIKSVRKLIEKLEKS[ENLYFQGLEHHHHHH] |
| R1-8 | MKEEQLKTFWEKVRHEFETLKLQLEQKGDPEIAKKIIEILKKWIEILKM YEKNADSPEIQKTVREYIERLKKWIEEMKKIA[ENLYFQGLEHHHHHH] |
| R1-9 | MKEEQLKLFWEKVRHEFETLKLQLEQKGDPEIAKKIIEILKKWIELLK MYEKNADSPEIQKTVREYIERLKKWIEEMKKIA[ENLYFQGLEHHHHH H] |
| R1-10 | MSMEEQLKTFYEKFKHEYEQLEALGVDKEQIKEWLKKFYKLFQMYE ENAETPEIQKLVRKYLKEIEKLLKELES[ENLYFQGLEHHHHHH] |
| R1-11 | MSEEEQAKTFEEKVRHEIEIALLQNVDPKELKKLKEEWVKLLRMYFG VSEEEARRLIEKWWREVEKSVRSK[ENLYFQGLEHHHHHH] |
| R2-1 | [MHHHHHHHHENLYFQGGS]SIKEMVRELQELFKEIEKKAEEALKKND PELLFRLMIKLQNLIIILMQYLKKANNPELVQMAVQVLYLLVQWLYE AYRKNDPEKMSRYIQELLEILKEILKIMEQL |
| R2-2 | [MHHHHHHHHENLYFQGGS]LEQELLELIQKYFKKWLKLIEELAEKND PESIMKLFEKALEILYKLIEMAKRLNTPFLVTVLLLAIFYFVQLFLILLK EGDPKKVLKVAKEYLKRLIELIEKYEKG |
| R2-3 | [MHHHHHHHHENLYFQGGS]RKEEMLREVYEIIQEYIKRAIELFQRGDP ESALKILQELMEILLRLMMKARKLGNPKILAFIVNVLKALHEAFLRLL QASTPEQAEKILKELLELVSRLFEYVQKS |

| | |
|---|---|
| R2-4 | [MHHHHHHHHENLYFQGGS]SEEEEAKKIAKEALEVAKRVEEYIRKND PESVKKLLKRIIELMRKAVKLAAKVNNPEVYVILINIIKLLHELFLLALR LGNPEIMEALYRALKVLMQLMFYFLKVV |
| R2-5 | [MHHHHHHHHENLYFQGGS]SLKELLQEIMKRIIEVLELIKEALDKNDP EALQKLFLELERMLEILMELAQKANNPIVVKIIIIMMKLVHEMFMIFIK EGDPKKIKEILEEMIQLAKKMIEILRKL |
| R2-6 | [MHHHHHHHHENLYFQGGS]SIKEELQELLKRLIELAELLKEYLDKNDP ESIQKILLEMERIAERVMELAQKANTPVVVKAIIIIMKLLHEMFMIFFKE GDPKKIKEILEEIIQILKKLIEYLQKA |
| R2-7 | [MHHHHHHHHENLYFQGGS]SLKELIQELLKQLEKILELLEEAADKND PEALQKLFLLFERILEQIMEYVQKANTPVLLKAIIEMMKIMHEMFEILI KEGDPEKIKKLLKILKELVRRMIEMVRKV |
| R2-8 | [MHHHHHHHHENLYFQGGS]EEQRLAQEFLEQLEEYIRRLIEALRKND KETFLKLVMKLEEWLLKWMQIFKRAGNTMLVVAVIIAMYILVQILLK WMRKNDVESAERMFEKLLKIVEKIVKEFQKK |
| R2-9 | [MHHHHHHHHENLYFQGGS]QIKEFAQKVAKKLIELFKKLEEAAKKN DPETFLKIVMEIEELLLKAMQFFKRAGNPELVVYIIILMYVLVQMLLIF MRENDPKKLKEYLELLQKLAKKMLEYAQKA |
| R2-10 | [MHHHHHHHHENLYFQGGS]QIKEFAQKVAKKLIELFKKLEEAAKKN DPETFLKIVMEIEELLLKAMQFFMKAGNPELLVYIITLMKVLHEMLYIF MQENDPKKLKEYLELLQKLAKKMLEYAQKA |
| R2-11 | [MHHHHHHHHENLYFQGGS]ELVEEVKELLKEFEKLVKLIIKLVQDGD PESAKEVAKRAKKIAKRMMKLLQKANTPEAYTVAIQMLYVAVQLAL IALEAGTPIAFEVAELLVKLAERVLLYLYRKL |
| R2-6.1 | [MGSSHHHHHHSSGENLYFQGHM]KEKERLQELLKRLIELAEQLKEAL EKGDPESIQKILTEMERIAEEVMELAQKANTPKVVKAIIEIMKLLHEMF DIFFKEGDPEKIKKILEEIIRILKELIEHLQKA |
| R2-6.2 | [MGSSHHHHHHSSGENLYFQGHM]KEKERLQELLKRLIELAEQLKEAL EKGDPESFQKILTEMERIAEEVMELAQKANTPKVVKAIIEIMKLLHEM FDIFHKGADPEEIKKILEEIIRILKELIEHLQKA |

**Table S2. Size-exclusion chromatography combined with multi-angle scattering data for the rescued designs.** The table provides the molecular weight ($M_w$) estimated from the main peak of each chromatogram in comparison with the theoretical value obtained from the amino acid sequence. Samples were run on a Superdex 75 10/300 increase and were prepared in PBS buffer. The close similarity between the theoretical and measured molecular weights confirms the monomeric state of the proteins.

| Design name | Theoretical $M_w$ (kDa) | Estimated Mw (kDa) |
|---|---|---|
| R2-6.1 | 15.0 | 16.9 (±6.3%) |
| R2-6.2 | 15.0 | 17.1 (±6.7%) |
